# Drug-Gene Interactions of Antihypertensive Medications and Risk of Incident Cardiovascular Disease: A Pharmacogenomics Study from the CHARGE Consortium

**DOI:** 10.1371/journal.pone.0140496

**Published:** 2015-10-30

**Authors:** Joshua C. Bis, Colleen Sitlani, Ryan Irvin, Christy L. Avery, Albert Vernon Smith, Fangui Sun, Daniel S. Evans, Solomon K. Musani, Xiaohui Li, Stella Trompet, Bouwe P. Krijthe, Tamara B. Harris, P. Miguel Quibrera, Jennifer A. Brody, Serkalem Demissie, Barry R. Davis, Kerri L. Wiggins, Gregory J. Tranah, Leslie A. Lange, Nona Sotoodehnia, David J. Stott, Oscar H. Franco, Lenore J. Launer, Til Stürmer, Kent D. Taylor, L. Adrienne Cupples, John H. Eckfeldt, Nicholas L. Smith, Yongmei Liu, James G. Wilson, Susan R. Heckbert, Brendan M. Buckley, M. Arfan Ikram, Eric Boerwinkle, Yii-Der Ida Chen, Anton J. M. de Craen, Andre G. Uitterlinden, Jerome I. Rotter, Ian Ford, Albert Hofman, Naveed Sattar, P. Eline Slagboom, Rudi G. J. Westendorp, Vilmundur Gudnason, Ramachandran S. Vasan, Thomas Lumley, Steven R. Cummings, Herman A. Taylor, Wendy Post, J. Wouter Jukema, Bruno H. Stricker, Eric A. Whitsel, Bruce M. Psaty, Donna Arnett

**Affiliations:** 1 Cardiovascular Health Research Unit, Department of Medicine, University of Washington, Seattle, Washington, United States of America; 2 Department of Epidemiology, University of Alabama at Birmingham, Birmingham, Alabama, United States of America; 3 Department of Epidemiology, University of North Carolina Gillings School of Global Public Health, Chapel Hill, North Carolina, United States of America; 4 Icelandic Heart Association, Kopavogur, Iceland; 5 Faculty of Medicine, University of Iceland, Reykjavik, Iceland; 6 Department of Biostatistics, Boston University School of Public Health, Boston, Massachusetts, United States of America; 7 California Pacific Medical Center Research Institute, San Francisco, California, United States of America; 8 Department of Medicine, University of Mississippi Medical Center, Jackson, Mississippi, United States of America; 9 Institute for Translational Genomics and Population Sciences, Los Angeles Biomedical Research Institute at Harbor-UCLA Medical Center, Torrance, California, United States of America; 10 Department of Cardiology, Leiden University Medical Center, The Netherlands; 11 Department of Gerontology and Geriatrics, Leiden University Medical Center, The Netherlands; 12 Department of Epidemiology, Erasmus Medical Center, Rotterdam, The Netherlands; 13 Laboratory of Epidemiology, Demography, and Biometry, National Institute on Aging, National Institutes of Health, Bethesda, Maryland, United States of America; 14 Collaborative Studies Coordinating Center, University of North Carolina Gillings School of Global Public Health, Chapel Hill, North Carolina, United States of America; 15 Department of Biostatistics, University of Texas School of Public Health, Houston, Texas, United States of America; 16 Department of Epidemiology and Biostatistics, University of California San Francisco, San Francisco, California, United States of America; 17 Department of Genetics, University of North Carolina, Chapel Hill, North Carolina, 27599, United States of America; 18 Cardiology Division, University of Washington, Seattle, Washington, United States of America; 19 Institute of Cardiovascular and Medical Sciences, Faculty of Medicine, University of Glasgow, Glasgow, United Kingdom; 20 University of North Carolina—GSK Center of Excellence in Pharmacoepidemiology, Chapel Hill, North Carolina, United States of America; 21 Department of Pediatrics, Harbor-UCLA Medical Center, Torrance, California, United States of America; 22 The Framingham Heart Study, Framingham, Massachusetts, United States of America; 23 Department of Laboratory Medicine and Pathology, University of Minnesota, Minneapolis, Minnesota, United States of America; 24 Department of Epidemiology, University of Washington, Seattle, Washington, United States of America; 25 Seattle Epidemiologic Research and Information Center of the Department of Veterans Affairs Office of Research and Development, Seattle, Washington, United States of America; 26 Department of Epidemiology and Prevention, Division of Public Health Sciences, Wake Forest University, Winston-Salem, North Carolina, United States of America; 27 Department of Physiology and Biophysics, University of Mississippi Medical Center, Jackson, Mississippi, United States of America; 28 Department of Pharmacology and Therapeutics, University College Cork, Cork, Ireland; 29 Institute for Molecular Medicine, University of Texas Health Science Center, Houston, Texas, United States of America; 30 Department of Internal Medicine, Erasmus Medical Center, Rotterdam, The Netherlands; 31 Robertson Center for Biostatistics, University of Glasgow, Glasgow, United Kingdom; 32 BHF Glasgow Cardiovascular Research Centre, Faculty of Medicine, Glasgow, United Kingdom; 33 Department of Molecular Epidemiology, Leiden University Medical Center, Leiden, The Netherlands; 34 Faculty of Health and Medical Sciences, Department of Public Health, University of Copenhagen, Copenhagen, Denmark; 35 Boston University School of Medicine, Boston, Massachusetts, United States of America; 36 Boston University School of Public Health, Boston, Massachusetts, United States of America; 37 Department of Statistics, University of Auckland, Auckland, New Zealand; 38 Department of Medicine, Morehouse School of Medicine, Atlanta, Georgia, United States of America; 39 Division of Cardiology, Department of Medicine, Johns Hopkins University, Baltimore, Maryland, United States of America; 40 Inspectorate for Health Care, the Hague, The Netherlands; 41 Department of Medicine, University of North Carolina School of Medicine, Chapel Hill, North Carolina United States of America; 42 Department of Health Services, University of Washington, Seattle, Washington, United States of America; 43 Group Health Research Institute, Group Health, Seattle, Washington, United States of America; University of Bologna, ITALY

## Abstract

**Background:**

Hypertension is a major risk factor for a spectrum of cardiovascular diseases (CVD), including myocardial infarction, sudden death, and stroke. In the US, over 65 million people have high blood pressure and a large proportion of these individuals are prescribed antihypertensive medications. Although large long-term clinical trials conducted in the last several decades have identified a number of effective antihypertensive treatments that reduce the risk of future clinical complications, responses to therapy and protection from cardiovascular events vary among individuals.

**Methods:**

Using a genome-wide association study among 21,267 participants with pharmaceutically treated hypertension, we explored the hypothesis that genetic variants might influence or modify the effectiveness of common antihypertensive therapies on the risk of major cardiovascular outcomes. The classes of drug treatments included angiotensin-converting enzyme inhibitors, beta-blockers, calcium channel blockers, and diuretics. In the setting of the Cohorts for Heart and Aging Research in Genomic Epidemiology (CHARGE) consortium, each study performed array-based genome-wide genotyping, imputed to HapMap Phase II reference panels, and used additive genetic models in proportional hazards or logistic regression models to evaluate drug-gene interactions for each of four therapeutic drug classes. We used meta-analysis to combine study-specific interaction estimates for approximately 2 million single nucleotide polymorphisms (SNPs) in a discovery analysis among 15,375 European Ancestry participants (3,527 CVD cases) with targeted follow-up in a case-only study of 1,751 European Ancestry GenHAT participants as well as among 4,141 African-Americans (1,267 CVD cases).

**Results:**

Although drug-SNP interactions were biologically plausible, exposures and outcomes were well measured, and power was sufficient to detect modest interactions, we did not identify any statistically significant interactions from the four antihypertensive therapy meta-analyses (P_interaction_ > 5.0×10^−8^). Similarly, findings were null for meta-analyses restricted to 66 SNPs with significant main effects on coronary artery disease or blood pressure from large published genome-wide association studies (P_interaction_ ≥ 0.01). Our results suggest that there are no major pharmacogenetic influences of common SNPs on the relationship between blood pressure medications and the risk of incident CVD.

## Introduction

Typically asymptomatic, hypertension is a major risk factor for several serious common clinical cardiovascular diseases (CVD), including myocardial infarction, sudden death, and stroke. In the US, over 65 million people have high blood pressure [1]. Large long-term clinical trials conducted in the last several decades have identified a number of effective treatments that reduce the risk of future clinical complications [2,3]. However, responses to therapy and protection from cardiovascular events vary among individuals. We hypothesized that underlying genetic variation might explain observed inter-individual differences in cardiovascular protection from major classes of antihypertensive medications. Identifying potential drug-gene interactions that affect the efficacy or safety of antihypertensive medications, particularly in relation to risk of CVD, is a first step in a translational research effort aimed to decrease the burden of a major public health problem.

Recent genome-wide association studies (GWAS) have identified a large number of common single nucleotide polymorphisms (SNPs) associated with blood pressure [2] and coronary artery disease [3]. To date, however, GWAS examining pharmacogenomics effects for antihypertensive therapies have tended to focus on the outcome of blood pressure [4, 5] or the adverse metabolic responses to antihypertensive drug treatments [6, 7] rather than on clinical events. Although the sample sizes have tended to be small, often less than 1000 participants, clever design features for validation [4] and high-fidelity phenotyping [8] have improved the yield from GWAS studies of drug-gene interactions on levels of blood pressure. For example, a study using a two stage GWAS design of angiotensin converting enzyme (ACE) activity identified a candidate gene subset for follow-up analyses of blood pressure. The results suggested an interaction on blood pressure response to ACE inhibitor monotherapy for carriers of *ACE* and *ABO* polymorphisms [9].

For the outcome of cardiovascular events, most published pharmacogenomics studies of drug-gene interactions have typically employed a candidate gene approach [10–12] or have evaluated modest-sized panels of SNPs [13]. Among evaluations of larger numbers of SNPs, a recent case-cohort study tested [12] common non-synonymous SNPs from the Illumina HumanCVD Beadchip within the INVEST trial, in which participants with coronary artery disease and hypertension were randomized to a beta-blocker or a calcium channel blocker, for SNP-by-treatment interaction on risk of all-cause death, nonfatal MI or nonfatal stroke. As follow-up, a gene score calculated from the top two variants from the discovery analysis–SNPs in *SIGLEC12* and *A1BG*–was associated with differences in risk among participants in the Nordic Diltiazem study who used calcium channel blockers [13].

We aimed to build upon prior research by more fully characterizing common variation across the entire genome and by focusing on population-based samples of treated hypertensive participants with long prospective follow-up for incident cardiovascular events. Thus, using genome-wide data on over 2 million measured and imputed SNPs from ongoing studies, this project represents a multi-center collaborative effort to identify major antihypertensive therapy-associated pharmacogenomics interactions associated with sudden death, myocardial infarction (MI) or stroke in patients with treated hypertension.

## Materials & Methods

### Design

The original design was a two-stage study of drug-gene interactions on the outcome of cardiovascular disease with discovery in a case-control study and replication among two cohort studies. The first stage was expected to have 1,500 cases and 2,600 controls, and the second stage was expected to have 1,250 CVD cases and 5,250 non-cases over the course of follow-up. All participants were treated for hypertension and cardiovascular disease (CVD) cases included myocardial infarction, coronary death, sudden death, and stroke. The proposed study had good power to detect modest-sized interactions with common genetic variants, for instance, 80% power to detect a multiplicative interaction of 2.3 for a variant with an allele frequency of 0.2 and a drug prevalence of 25%.

During the conduct of the study, collaborations with other cohort studies and clinical trials provided an opportunity to expand the European ancestry sample size to include 3,527 CVD cases and 11,848 controls in the discovery stage and 1,751 CVD cases in the case-only replication stage. To limit genotyping costs, the design remained two-stage. Across the two stages, our study had good power to detect modest-sized interactions with common genetic variants, for instance, 97% power to detect a multiplicative interaction effect of 1.6 for a variant with an allele frequency of 0.2 and a drug prevalence of 25%.

### Study population

We performed a discovery meta-analysis (“Stage I”) of nine studies with GWA data that included participants of European ancestry (EA) with treated hypertension in the setting of the Cohorts for Heart and Aging Research in Genomic Epidemiology (CHARGE) consortium [14]: the Age, Gene/Environment Susceptibility–Reykjavik Study (AGES), the Atherosclerosis Risk in Communities study (ARIC), the Cardiovascular Health Study (CHS), the Framingham Heart Study (FHS), the Health, Aging, and Body Composition (Health ABC) study, the Heart and Vascular Health Study (HVH), the Multi-Ethnic Study of Atherosclerosis (MESA), the Prospective Study of Pravastatin in the Elderly at Risk (PROSPER), and the Rotterdam Study (RS). Details of the individual studies are available in the **Supplementary Material (Section A in S1 File)**. Among these samples, we also explored variants that had demonstrated significant results on the main effects of systolic or diastolic blood pressure [2] and coronary artery disease [3] in other published genome-wide association studies.

We then selected variants with the lowest p-values in Stage I for genotyping in Stage II among European ancestry participants from the Genetics of Hypertension Associated Treatment (GenHAT) ancillary case-only study of the ALLHAT clinical trial [15, 16]. Briefly, ALLHAT was a randomized, double-blind, multicenter clinical trial of hypertensive adults designed to determine if the incidence of fatal CHD and nonfatal myocardial infarction was lower among patients randomized to one of four antihypertensive drug classes: a calcium channel blocker (amlodipine), an ACE inhibitor (lisinopril), and an alpha-adrenergic blocker (doxazosin), each compared with a diuretic (chlorthalidone). The case-only phase of GenHAT focused on discovering pharmacogenetic associations with candidate genes among 11,599 ALLHAT participants who experienced an adverse event (fatal CHD or nonfatal myocardial infarction, stroke, heart failure, coronary revascularization, angina, peripheral arterial disease, end-stage renal disease, all-cause death).

Although we did not have power for discovery analyses among non-European ancestry participants, we nevertheless conducted exploratory analyses to evaluate evidence for antihypertensive pharmacogenomic effects among African-American (AA) participants from four of the discovery studies (ARIC, CHS, Health ABC, MESA), as well as GenHAT and the Jackson Heart Study (JHS) and to provide additional context to our discovery findings. Each study followed a pre-specified analysis protocol and findings from the within-study analyses were then combined via meta-analysis, as described below.

### Ethics Statement

All studies were approved by local ethics committees and all participants provided written informed consent. The ethics committees for the individual studies are: AGES: The National Bioethics Committee, Iceland; ARIC: University of North Carolina at Chapel Hill Office of Human Research Ethics; CHS: University of Washington Human Subjects Division IRB; FHS: Institutional Review Board of Boston University Medical Campus and Boston Medical Center; GenHAT: UAB Institutional Review Board for Human Use (IRB); Health ABC: clinic protocols were approved by the University of Pittsburgh Institutional Review Board and the University of Tennessee Health Science Center Institutional Review Board, and the Coordinating Center protocols were approved by the UCSF Human Research Protection Program/Committee on Human Research; HVH: Group Health Cooperative Human Subjects Review Committee; JHS: The Institutional Review Board of the University of Mississippi Medical Center; MESA: Los Angeles Biomedical Research Institute at Harbor-UCLA Human Subjects Institutional Review Board; PROSPER: Research Ethics Committee of the Cork Teaching Hospitals (CREC), Scottish Multi-Regional Ethics Committee A, and Medical Ethical Committee (METc) of the Leiden University Medical Center Rotterdam Study: Medical Ethics Committee of the Erasmus Medical Center.

### Study participants

This study was restricted to participants with treated hypertension, defined by the use of antihypertensive medications. Participants in longitudinal cohort studies entered the cohort of treated hypertensives at the examination when antihypertensive medications were first recorded and remained in the analysis for all observations in which they were users of antihypertensives. Participants with prevalent CVD at study baseline or prior to the initiation of HTN medication were excluded.

### Definition of drug exposure

We examined four therapeutic classes of drugs: ACE inhibitors, beta blockers, calcium channel blockers, and thiazide diuretics. Drug groupings were based on manually-curated lists that were reviewed by experts from each study to include all relevant drugs from the U.S. and Europe. Participants were classified as thiazide users if they took a thiazide or thiazide-like diuretic in a single or combination preparation, with or without potassium sparing diuretic or potassium supplements. Calcium channel blockers included both long- and short-acting preparations. For primary analyses, angiotensin receptor blockers (ARBs) were grouped with ACE inhibitors.

Drug exposures were assessed by medication inventory or self-report (prospective cohorts), computerized databases (HVH), or through randomized treatment assignment updated by self-report during follow-up (GenHAT). For each analysis, users of a given class were treated as the exposed group, users of all other classes served as the reference group. In this way, participants who used multiple classes of drugs appeared in multiple analyses and users were allowed to change drug classes during the course of follow-up, approximating an as-treated analysis.

### Definition of CVD outcome

The primary outcome of the study was incident CVD, which included myocardial infarction, coronary death, sudden death, or stroke. In secondary analyses, we considered an endpoint limited to incident MI events. Details of event definitions and surveillance methods for each of the participating studies are provided in **Supplementary Material (Section B in S1 File)**.

### Genotype arrays and imputation

Genome-wide SNP genotyping was performed within each cohort using Illumina or Affymetrix genotyping arrays. Follow-up (“Stage II”) genotyping of selected variants was performed using a custom content on the Illumina Human Exome array. Details of genotyping and QC are provided in Supplementary Material (Section C in S1 File); these procedures generally involved exclusion of participants on the basis of sex mismatches and duplicate samples; limitation to unrelated individuals in all cohorts except the family-based FHS; exclusion of samples with genotyping success rate <95%; and exclusion of SNPs failing genotyping call rate thresholds, typically between 95% and 99%.

To increase coverage and facilitate evaluation of the same SNPs across cohorts, SNPs passing quality control were used to impute to the HapMap Phase 2 reference panels using MaCH [17], BEAGLE, [18] or BIMBAM [19].

### Statistical analysis

#### Stage I Analysis

For the discovery analyses, each study performed four analyses of incident CVD across approximately 2 million autosomal SNPs. For the Stage I analyses, depending on individual study design and availability of follow-up data, studies evaluated drug-gene interactions in one of two ways: (1) Cox proportional hazards regression models with time-varying antihypertensive medication exposures in which participants entered the risk set at the first known report of antihypertensive treatment, remained in the risk set for all observations in which they were users of antihypertensive medications (i.e., those who stopped could re-enter upon re-initiation), and were followed until their first CVD event, death, or at the date of last follow-up; (2) Logistic regression in which all hypertensive CVD cases were compared to an age and sex-matched set of population-based hypertensive controls. [10]

All regression models included an additive SNP×drug interaction term and were adjusted for age, sex, recruitment site (when appropriate), and principal components for global ancestry (as needed); family based studies additionally adjusted for relatedness. Details of the software packages used to estimate cohort-specific results are shown in **Supplementary Materials (Section D in S1 File)**.

Study-specific interaction estimates (β) and standard errors (SE) were combined by fixed effects inverse variance weighted meta-analysis using METAL. [20] To control inflation for poorly-calibrated tests for less common variants among less common drug exposures, we first calculated SNP-specific degrees of freedom for each cohort as the product of the number of drug-exposed participants, the number of CVD events, the rate of drug exposure, the SNP imputation quality (range: 0, 1), and the minor allele frequency (MAF) (range: 0, 0.50). We excluded cohort-specific results for SNPs with fewer than 10 degrees of freedom. Per-study, we omitted SNPs from the meta-analysis if estimates were only available from two or fewer contributing studies or if the absolute value of the β estimate for interaction was greater than 5.0, indicating non-convergence of the regression. Finally, study-specific results were corrected by their respective genomic inflation factors (λ_gc_) before meta-analysis according to the genomic control method. [21] The genome-wide threshold for significant drug-SNP interaction was P < 5.0×10^−8^.

#### Stage II Analysis

We used p-values from the Stage I discovery meta-analysis to select approximately 200 high signal markers for each of the three classes of hypertension drugs tested in the ALLHAT trial (ACE inhibitors, diuretics, and calcium-channel blockers). Because beta-blockers were not a randomization group for ALLHAT (and instead were the default second-line agent), we did not pursue discovery findings from this drug class in the GenHAT case-only study. When multiple SNPs clustered at a single locus, we “trimmed” the list of SNPs on the basis of linkage disequilibrium (r^2^ < 0.7). Using a custom-content Illumina HumanExome Chip, we then attempted to genotype these signal SNPs, along with proxies selected on the basis of GWAS associations (discovery p-value within 10X of the signal marker) and 1000 Genomes linkage disequilibrium patterns.

The case-only GenHAT study estimated drug-gene interactions by modeling genotype as the predictor and drug exposure as the outcome [22, 23], a modeling strategy that assumes that genotype is not associated with choice of medication, which is a weaker assumption in ALLHAT because of the randomization.

Limiting to one SNP per locus (either lead or proxy, depending on Stage II availability), results from the Stage II case-only analyses were combined with the Stage I meta-analysis results in a fixed-effects inverse variance meta-analysis using METAL.

#### Analysis among African-Americans

We further explored top associations from the Stage I meta-analysis among African-American participants from ARIC, CHS, Health ABC, MESA, JHS and GenHAT.

## Results

We performed genome-wide association analyses across 15,375 EA participants from 9 studies to examine whether common genetic variants modified the associations between four common antihypertensive therapies and the risk of incident CVD (3,527 cases of the composite CVD outcome; 2,114 of which were cases of myocardial infarction) with targeted follow-up in a case-only study of 1,751 European Ancestry GenHAT participants as well as among 4,141 African-Americans (1,267 CVD cases). Characteristics of 21,267 study participants are shown in **Table 1** and **Tables A & B in S1 File**. On average, participants were predominantly female and middle-aged (mean age range = 56–77 years). The estimated prevalence of drug exposure at study baseline among the EA discovery sample was highest for diuretics and lowest for calcium channel blockers (**Table 1, Table C in S1 File**). Approximately 2 million autosomal SNPs were available for analysis after applying genotyping, imputation, and analytic quality control measures (**Table D in S1 File**). Q-Q plots based on meta-analyses of the cohort-specific, drug-SNP interaction parameters revealed little evidence of inflation of p-values demonstrated by lambdas less than 1.0 (**Figure A in S1 Figs**).

**Table 1:** Characteristics of Study Participants.

**Stage I**															
	**Model**	**Cases, N**	**Non-Cases, N**	**Age, y**		**Female, %**		**ACE, %**		**BB, %**		**CCB, %**		**Diuretics, %**	
AGES	C	236	1267	76.7		63%		22%		57%		29%		43%	
ARIC	C	213	2313	58.1		54%		21%		57%		29%		43%	
CHS	C	439	1430	71.8		65%		32%		29%		29%		48%	
Health ABC	C	128	755	75.5		51%		52%		36%		39%		36%	
MESA	C	66	874	66.4		51%		50%		35%		25%		48%	
		**Cases, N**	**Controls, N**	**Cases**	**Controls**	**Cases**	**Controls**	**Cases**	**Controls**	**Cases**	**Controls**	**Cases**	**Controls**	**Cases**	**Controls**
FHS	L	132	660	67.4	66.4	36%	36%	41%	40%	36%	34%	31%	22%	30%	29%
HVH-1	L	1285	1005	66.4	65.9	45%	38%	39%	37%	36%	35%	22%	19%	35%	40%
HVH-2	L	381	682	64.2	65.1	40%	38%	42%	45%	43%	36%	16%	18%	40%	45%
PROSPER	L	406	2621	75.7	75.3	48%	64%	22%	20%	36%	34%	38%	30%	57%	57%
RS	L	241	241	77.6	75.9	59%	59%	36%	38%	53%	56%	27%	23%	30%	38%
**Stage II–Case Only**															
	**Mode l**	**Cases, N**	**Controls, N**	**Age, y**		**Female, %**		**ACE, %**		**BB, %**		**CCB, %**		**Diuretics, %**	
				**Cases**	**Controls**	**Cases**	**Controls**	**Cases**	**Controls**	**Cases**	**Controls**	**Cases**	**Controls**	**Cases**	**Controls**
GenHAT	L	1751	n/a	69.8	n/a	32%	n/a	27%	n/a	n/a	n/a	28%	n/a	47%	n/a
**African-American Extension**															
	**Model**	**Cases, N**	**Non-Cases, N**	**Age, y**		**Female, %**		**ACE, %**		**BB, %**		**CCB, %**		**Diuretics, %**	
ARIC	C	105	954	55.7		68%		24%		22%		23%		47%	
CHS	C	112	385	72.2		68%		32%		20%		44%		50%	
Health ABC	C	86	621	74.3		62%		46%		24%		45%		45%	
MESA	C	42	846	64.5		56%		46%		26%		38%		57%	
		**Cases, N**	**Controls, N**	**Cases**	**Controls**	**Cases**	**Controls**	**Cases**	**Controls**	**Cases**	**Controls**	**Cases**	**Controls**	**Cases**	**Controls**
JHS	L	34	68	60.7	60.6	67%	67%	68%	12%	27%	52%	50%	34%	44%	53%
GenHAT	L	888	n/a	68.8	n/a	47%	n/a	30%	n/a	n/a	n/a	26%	n/a	48%	n/a

Age indicates mean age. Model indicates analysis method: C, Cox proportional hazards regression; L, logistic regression. For prevalence of antihypertensive medication use ACE indicates Angiotensin-converting enzyme inhibitor (or angiotensin receptor blocker); BB, beta-blocker; CCB, calcium channel blocker; Diuretics, thiazide diuretics.For studies analyzed with logistic regression, summaries are provided separately for cases and controls.

**Fig 1:**
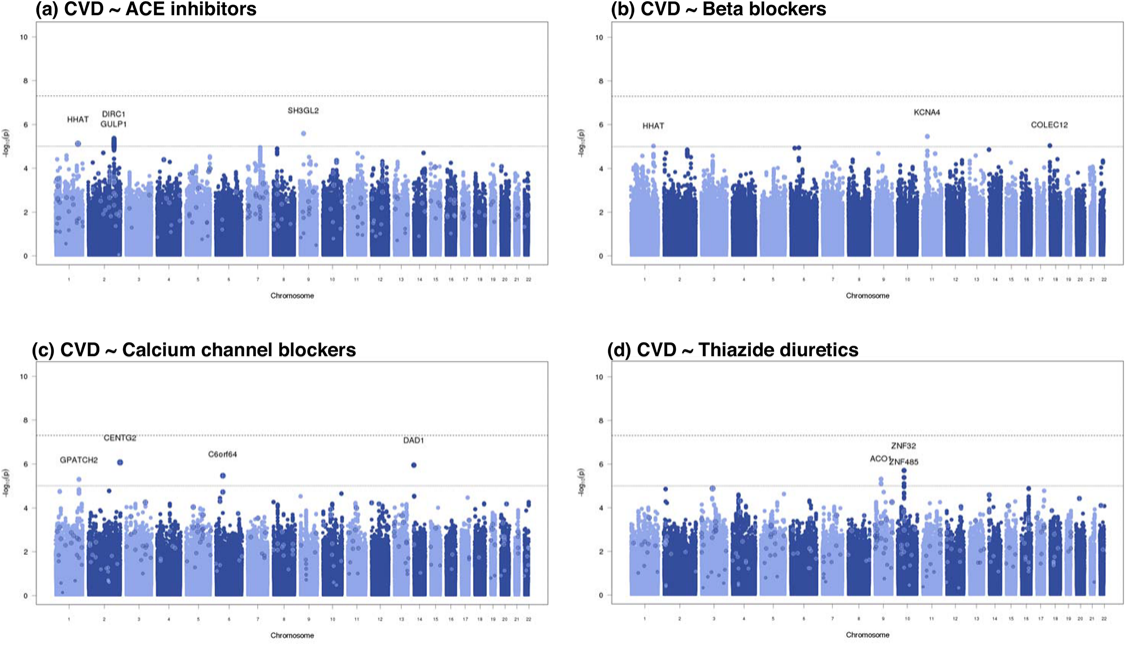
Plots show the individual interaction p-values based on Stage I (indicated as solid dots) or Stage I + Stage II meta-analysis (indicated as outlined dots with “+” symbol) against their genomic position for the combined cardiovascular disease (CVD) outcome for the four antihypertensive medication exposures: (a) Angiotensin-converting enzyme (ACE) inhibitors, (b) Beta-blockers, (c) Calcium Channel Blockers, and (d) Thiazide Diuretics. Within each chromosome, shown on the x-axis, the results are plotted left to right from the p-terminal end. The nearest genes are indicated for variants with an interaction p-value less than 1×10^−5^ in the meta-analysis.

We did not detect any genome-wide significant interactions (P < 5.0×10^−8^) for any of the four drug classes in Stage I (**Figure B in S1 Figs**). Thus the originally planned case-only replication effort became a second stage of discovery. However, when we combined results from Stage I and the 1,751 EA GenHAT CVD cases from Stage II, no SNP×drug interaction estimates attained statistical significance (p < 5.0×10^−8^) (**Fig 1, Table E in S1 File**). Further, the top ten loci from the combined Stage I and Stage II analyses showed no evidence of significance among the 4,141 African-American participants from Stage I and II (**Table F in S1 File**) combined. When we repeated the analyses limited to MI (rather than combined CVD) as an outcome among the EA sample, the results were similarly null (**Table G in S1 File**, **Figures C & D in S1 Figs**). Because beta-blockers were not a first line agent in GenHAT; we did not perform a combined Stage I & Stage II meta-analysis; however, no results attained statistical significance in Stage I (**Table H in S1 File**).

Of the 75 well-validated GWAS variants for coronary artery disease (n = 46) and blood pressure (n = 29), 66 were directly available in our Stage I meta-analysis. Meta-analyses restricted to these 66 SNPs were similarly null (interaction P > 0.0001 [0.05/264 tests], **Tables I & J in S1 File**).

## Discussion

In this study with a combined sample size of 17,126 European ancestry participants from 10 studies, we evaluated evidence for drug-gene interactions that influenced risk of incident CVD among participants treated for hypertension. Although we were well powered to discover modest interactions for common variants, we did not identify any genetic variants that significantly modified the association of antihypertensive medications with the risk of CVD in this large population-based sample of participants. Further characterization of top associations from the EA analyses among 4,141 African-Americans from 6 studies, a group with generally higher HTN medication usage and higher CVD event rates, did not reveal any significant associations.

Further, in addition to performing a genome-wide association study evaluating potential drug-gene interactions, we separately evaluated interactions for 66 SNPs previously associated with coronary artery disease or blood pressure main effects. Even using a less stringent significance threshold, we found that no previously identified SNPs modified the association between blood pressure lowering medications and the risk of CVD. This null result is perhaps not surprising, as SNPs selected on the basis of an extreme p-value for a single main effect may be less likely to harbor heterogeneity across population subgroups.

The strengths of the project include: the large size of the case group; the population-based designs for both the case-control and the cohort studies; and the high-quality ascertainment of events and medication status.

We chose a well-measured phenotype with biologically plausible pharmacogenomic effects, and our drug assessment methods were sensitive and reliable [24, 25], yet we were unable to detect any genome-wide significant interactions.

Our study was well powered for common variants and modest-to-large interactions, but we remained underpowered to detect smaller interactions; thus false negative findings that failed to meet stringent genome-wide significance thresholds are also possible, in particular for rarer alleles and less common exposures. Our analyses focused on common variants; so it is possible that areas of the genome and types of variants not well covered by GWAS methods, such as rare variants, may have been missed by this approach. Because common variants tend to be old and global, we are likely to have missed recent, local, and rare variants responsible for drug-gene interactions.

The other limitations are those of observational studies, including the possibility of confounding, selection bias, missing data, and population stratification or admixture. The clinical trial analogue of the case-control and cohort studies is the on-treatment rather than the intention-to-treat analysis. Confounding from unmeasured or unknown factors or measurement error in known risk factors remain alternative explanations for findings of all observational studies [26, 27]. In particular, because medication use was ascertained through prescription drug records or medication inventories, some level of exposure misclassification is possible. Further, our approach compared users of one drug class to a reference group that included users of three other classes and did not specifically account for simultaneous use of multiple therapies.

While our study considered interactions between common antihypertensive medications and variants across the genome, other strategies to investigate genetic influences on blood pressure treatment have been employed with varying levels of success. For instance, the CHARGE consortium investigated 30 candidate genes that code for proteins that are direct targets of antihypertensive drugs and found that only a few (*ADRB1*, *AGT*, *ACE*) had significant, modest associations with blood pressure levels (< 1mm Hg) or with hypertension (<10% difference in risk) [28]. Other studies have evaluated hypertensive susceptibility loci for potential effects on response to antihypertensive drug therapies [29]. By largely focusing on the outcome of blood pressure, the GWAS approach to antihypertensive drug-gene interactions has identified interactions of small to modest effect sizes [4, 5, 8, 9].

Our study focused on the major disease endpoints of MI, stroke and sudden death among treated hypertensive patients rather than the levels of blood pressure, primarily because these devastating clinical events are complications of hypertension. Because blood-pressure differences in trials of drug therapy do not translate directly into differences in the risks of clinical outcomes [30], we preferred to focus on cardiovascular events such as MI, stroke, and sudden death as the primary outcomes of interest. Indeed, before screening for the relevant genetic variants could be recommended, the drug-gene effects on blood pressure would need to be tracked from the surrogate endpoint of blood pressure to the prevention of major disease endpoints [31]. In an effort to examine a less heterogeneous outcome, we limited endpoints to MI cases in secondary analyses, which were also null. However, we did not have sufficient power to investigate stroke as its own outcome. Larger sample sizes will be necessary to examine pharmacogenetic results specific to stroke and blood-pressure lowering medications.

For many patients, medications offer substantial benefits that can be maximized by avoiding medications in patients who are susceptible to complications or by targeting particular medications to patients who are likely to benefit. The results presented here provide little evidence that common variant alleles modify the effect of frequently-prescribed hypertension therapies on the outcomes of MI, stroke and sudden death.

Although these findings do not illuminate new pathways in the risk of cardiovascular events, these null results nevertheless provide important information from the point of view of public health: our evidence suggests that there are no large common pharmacogenomic effects of blood pressure medications on CVD health outcomes; thus at this time genetic screening to guide choice of blood pressure therapy is not necessary. In conclusion, additional efforts to assemble even larger sample sizes and to more fully interrogate the human genome may be required to realize the potential of pharmacogenomics for antihypertensive drug treatment.

## Supporting Information

S1 Figs Supplementary Figures. (PDF)

S1 File Supplementary Materials. (PDF)
