# Supplementary material for: Drug-Gene Interactions of Antihypertensive Medications and Risk of Incident Cardiovascular Disease: A Pharmacogenomics Study from the CHARGE Consortium
Source: PLoS One. 2015 Oct 30;10(10):e0140496. doi: 10.1371/journal.pone.0140496 (PMC4627813; doi:10.1371/journal.pone.0140496)
Supplement: S1 Figs — (PDF) [file pone.0140496.s001.pdf]

Drug-gene interactions of antihypertensive medications and risk of incident cardiovascular disease: a pharmacogenomics study from the CHARGE consortium (Supplementary Figures)

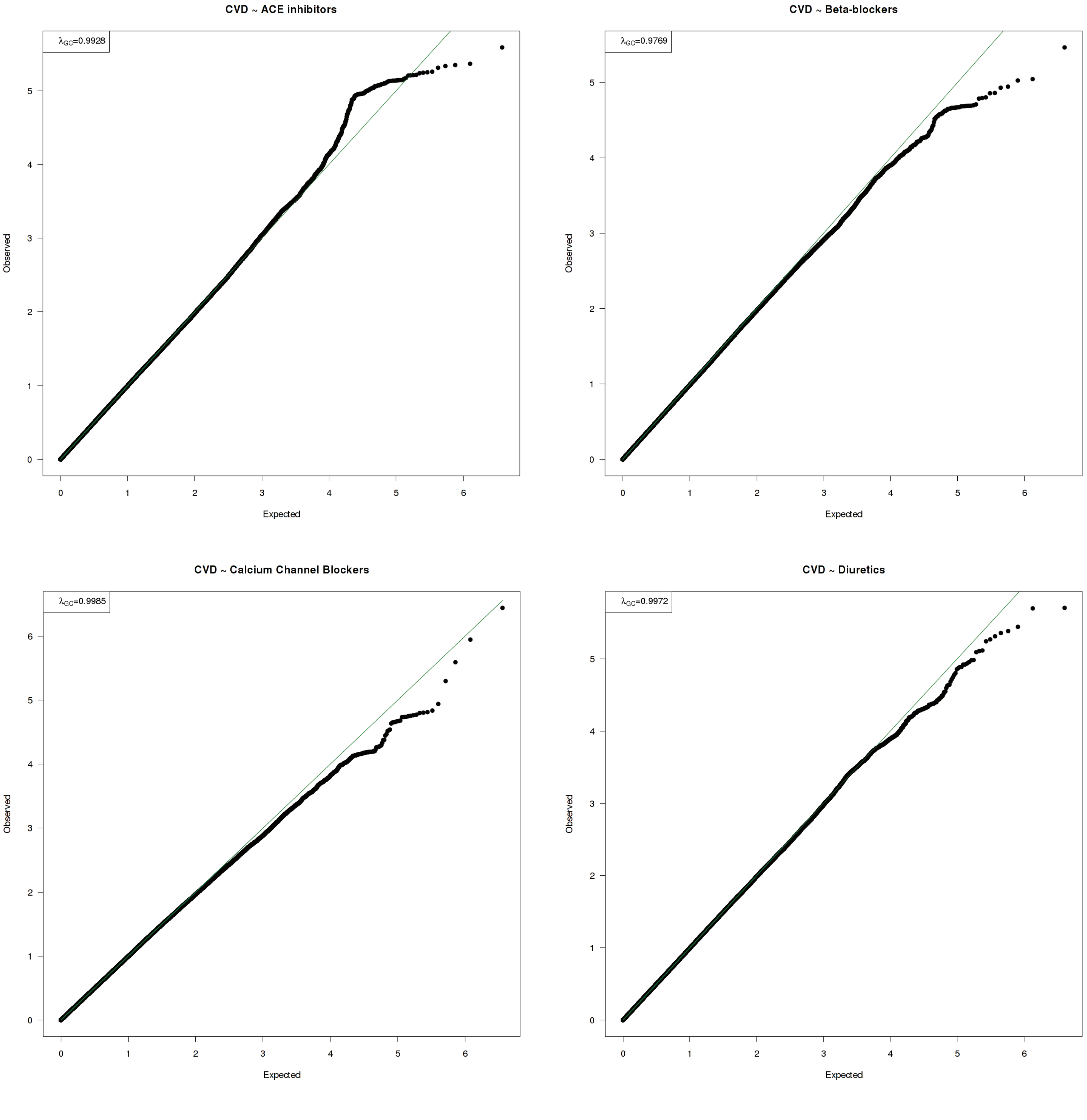

Supplementary Figure A: Q-Q plots for discovery meta-analyses for combined CVD outcome for the four antihypertensive medication exposures: Angiotensin-converting enzyme (ACE) inhibitors, Beta-blockers, Calcium Channel Blockers, and Thiazide Diuretics. λ_{GC} indicates genomic inflation factor.

Drug-gene interactions of antihypertensive medications and risk of incident cardiovascular disease: a pharmacogenomics study from the CHARGE consortium (Supplementary Figures)

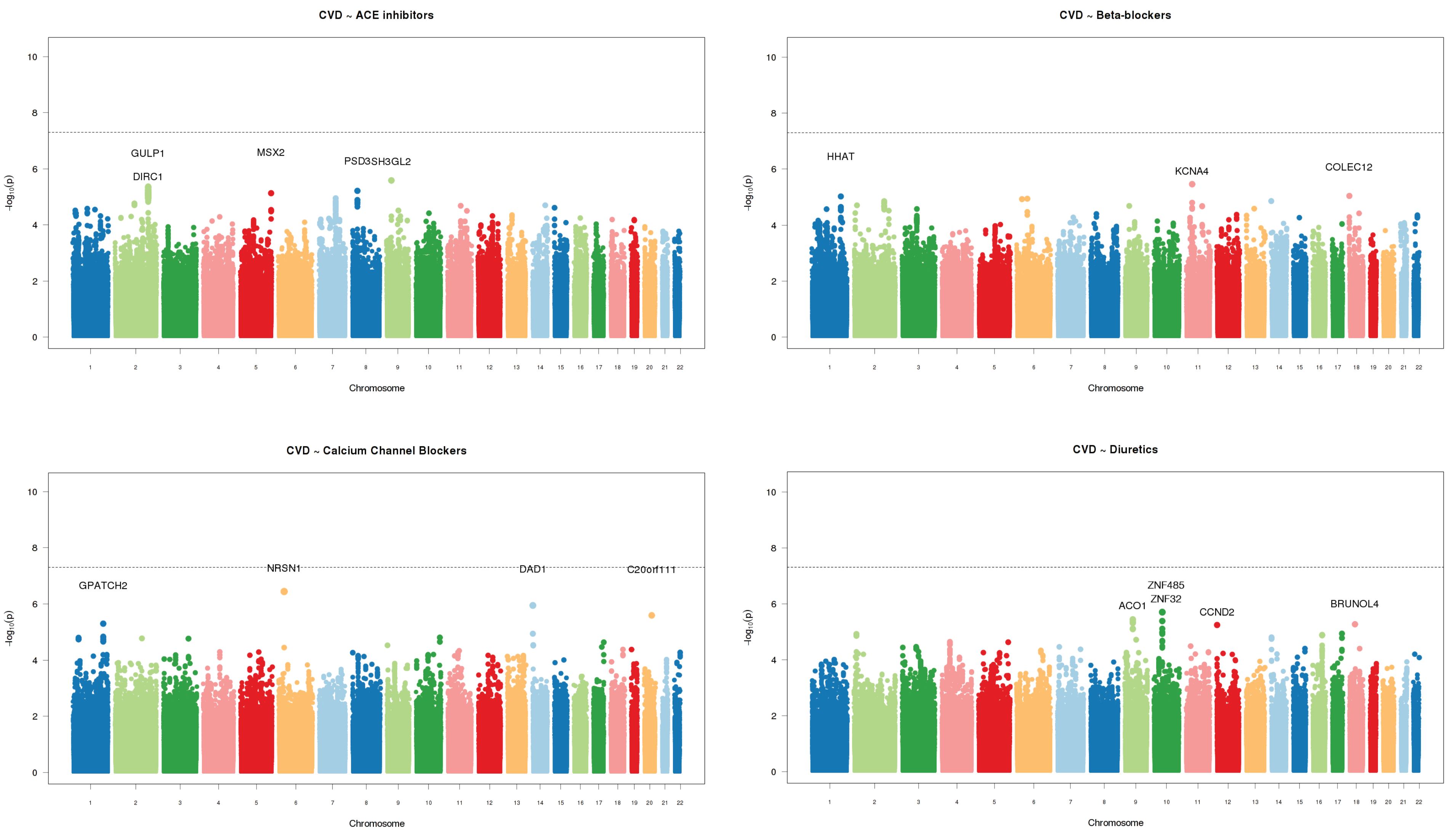

Supplementary Figure B: Plots show the individual interaction p-values based on discovery meta-analysis against their genomic position for Myocardial Infarction (MI) outcome for the four antihypertensive medication exposures: Angiotensin-converting enzyme (ACE) inhibitors, Beta-blockers, Calcium Channel Blockers, and Thiazide Diuretics. Within each chromosome, shown on the x-axis, the results are plotted left to right from the p-terminal end. The nearest genes are indicated for variants with an interaction p-value less than 1×10^{-5} in the discovery meta-analysis.

Drug-gene interactions of antihypertensive medications and risk of incident cardiovascular disease: a pharmacogenomics study from the CHARGE consortium (Supplementary Figures)

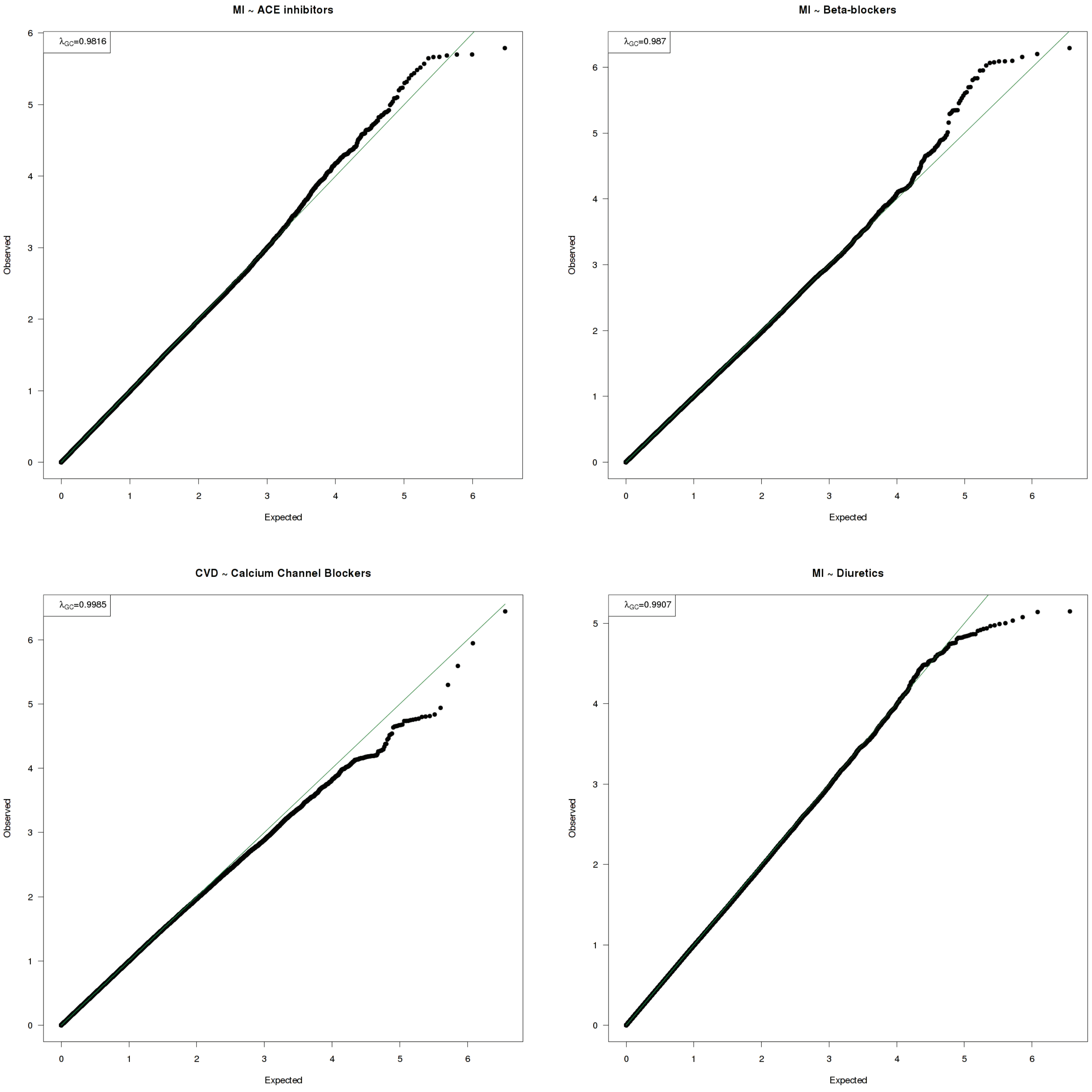

Supplementary Figure C: Q-Q plots for discovery meta-analyses for myocardial infarction (MI) outcome for the four antihypertensive medication exposures: Angiotensin-converting enzyme (ACE) inhibitors, Beta-blockers, Calcium Channel Blockers, and Thiazide Diuretics. λ_{GC} indicates genomic inflation factor.

Drug-gene interactions of antihypertensive medications and risk of incident cardiovascular disease: a pharmacogenomics study from the CHARGE consortium (Supplementary Figures)

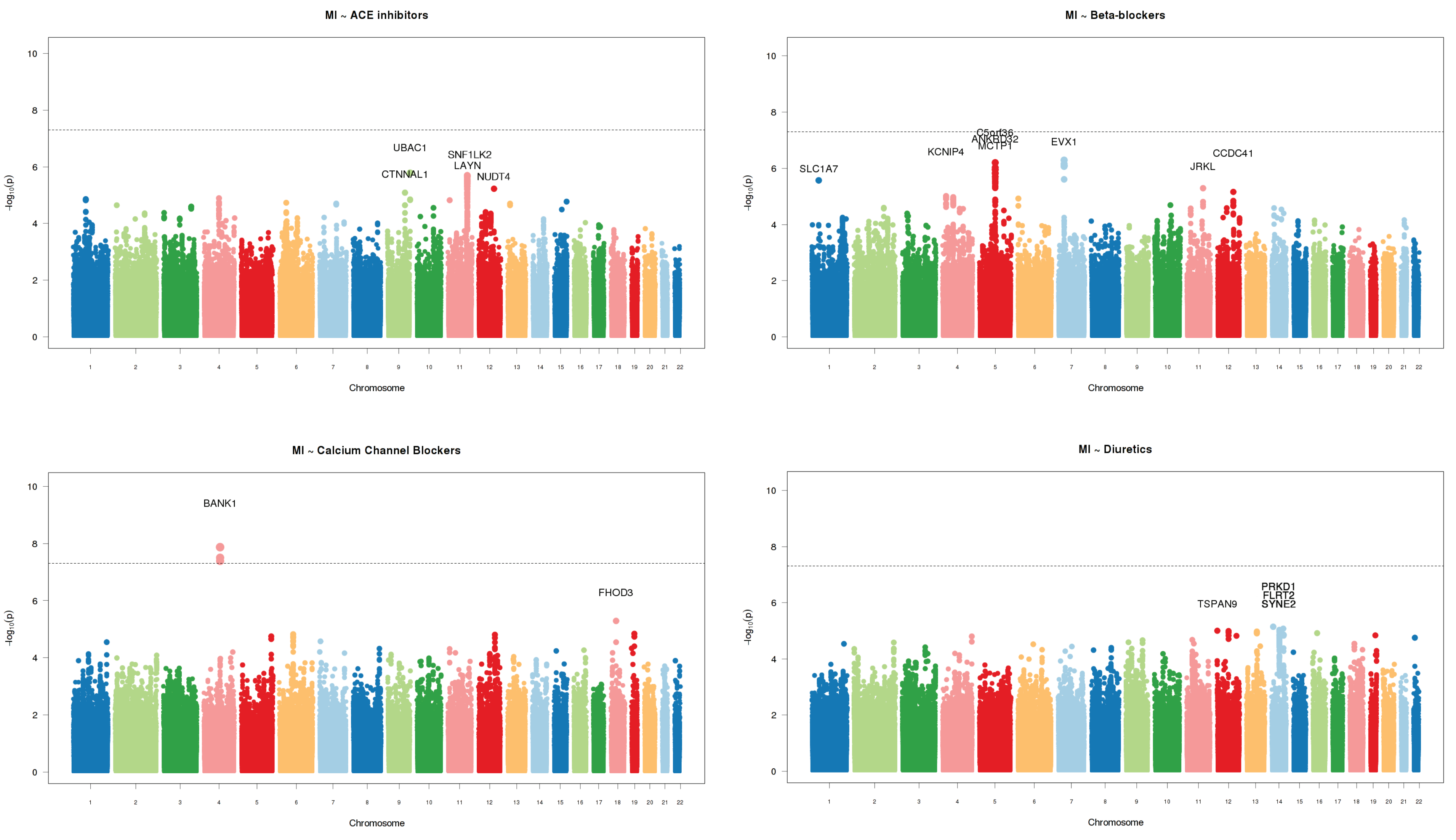

Supplementary Figure D: Plots show the individual interaction p-values based on discovery meta-analysis against their genomic position for Myocardial Infarction (MI) outcome for the four antihypertensive medication exposures: Angiotensin-converting enzyme (ACE) inhibitors, Beta-blockers, Calcium Channel Blockers, and Thiazide Diuretics. Within each chromosome, shown on the x-axis, the results are plotted left to right from the p-terminal end. The nearest genes are indicated for variants with an interaction p-value less than 1×10^{-5} in the discovery meta-analysis.
