# Supplementary material for: Drug-Gene Interactions of Antihypertensive Medications and Risk of Incident Cardiovascular Disease: A Pharmacogenomics Study from the CHARGE Consortium
Source: PLoS One. 2015 Oct 30;10(10):e0140496. doi: 10.1371/journal.pone.0140496 (PMC4627813; doi:10.1371/journal.pone.0140496)
Supplement: S1 File — (PDF) [file pone.0140496.s002.pdf]

Drug-gene interactions of antihypertensive medications and risk of incident cardiovascular disease: a pharmacogenomics study from the CHARGE consortium (Supplementary Materials)

Supplementary Table A: Characteristics of European Ancestry Participants, Stage I & II................2

Supplementary Table B: Characteristics of African-American Participants 2

Supplementary Table C: Prevalence of antihypertensive medication use...3

Supplementary Table E: Top associations for SNP×anti-hypertensive interactions on incident CVD risk from Stage I & Stage II meta-analysis 5

Supplementary Table F: Top associations for SNP×anti-hypertensive interactions on incident CVD risk from Stage I & Stage II meta-analysis among African-American Participants 6

Supplementary Table G: Top associations for SNP×anti-hypertensive interactions on incident MI risk from Stage I & Stage II meta-analysis 7

Supplementary Table H: Top associations for SNP×beta-blocker interactions on incident CVD risk from Stage I meta-analysis 8

Supplementary Table I: Interaction of Coronary Artery Disease GWAS SNP×antihypertensive use interactions on incident CVD risk from Stage I meta-analysis 9

Supplementary Table J: Interaction of Blood Pressure GWAS SNP×antihypertensive use interactions on incident CVD risk from Stage I meta-analysis 10

Supplementary Table A: Characteristics of European Ancestry Participants, Stage I & II

Stage I

Analyzed with Cox Proportional Hazards Regression

	CVD			MI			
	Cases, N	Non-Cases, N	Follow-up, y	Cases,N	Non-Cases, N	Age, y	Female, %
AGES	236	1267	5.59	96	1267	76.7 (5.4)	63.0%
ARIC	213	2313	7.52	160	2383	58.1 (6.3)	54.0%
CHS	439	1430	8.21	169	1700	71.8 (4.9)	65.3%
Health ABC	128	755	7.77	49	834	75.5 (4.0)	51.0%
MESA	66	874	3.54	41	899	66.4 (9.6)	50.7%

Analyzed with Logistic Regression

	CVD		MI		Age, y		Female, %	
	Cases, N	Controls, N	Cases,N	Controls, N	Case	Control	Case	Control
FHS	132	660	83	415	67.4 (8.5)	66.4 (8.4)	36.0%	35.9%
HVH-1	1285	1005	830	1005	66.4 (9.5)	65.9 (9.0)	45.4%	38.3%
HVH-2	381	682	295	682	64.2 (9.2)	65.1 (8.9)	40.4%	38.1%
PROSPER	406	2621	308	2621	75.7 (3.4)	75.3 (3.4)	48.0%	64.0%
RS	241	241	83	241	77.6 (7.7)	75.9 (7.3)	59.3%	59.3%

Stage II

	CVD			MI			
	Cases, N	Controls, N	Follow-up, y	Cases, N	Controls, N	Age, y	Female, %
GenHAT	1751	n/a	2.17	1171	n/a	69.8 (7.6)	32%

Numbers in table for age are mean and standard deviation; N indicates sample size; y, years;

Supplementary Table B: Characteristics of African-American Participants

Analyzed with Cox Proportional Hazards Regression

	CVD				
	Cases, N	Non-Cases, N	Follow-up, y	Age, y	Female, %
ARIC	105	954	7.30	55.7 (6.2)	68.0%
CHS	112	385	8.14	72.2 (5.2)	68.0%
Health ABC	86	621	7.87	74.3 (3.6)	62.0%
MESA	42	846	3.93	64.5 (9.4)	55.8%
GenHAT	888	n/a	2.27	68.8 (8.4)	47%

Analyzed with Logistic Regression

	CVD		Age, y		Female, %	
	Cases, N	Controls, N	Cases	Controls	Cases	Controls
JHS	34	68	60.7 (11.9)	60.6 (12.0)	66.7%	66.7%

Numbers in table for age are mean and standard deviation.

Supplementary Table C: Prevalence of antihypertensive medication use

Stage I										
Analyzed with Cox Proportional Hazards Regression										
	Sample Size, N		ACE, %		BB, %		CCB, %		Diuretics, %	
AGES	1503		21.5%		57.3%		28.9%		43.3%	
ARIC	2526		21.0%		57.0%		29.0%		43.0%	
CHS	1869		32.0%		29.0%		29.0%		48.0%	
Health ABC	883		52.0%		36.0%		39.0%		36.0%	
MESA	940		50.2%		34.7%		25.1%		48.2%	
Analyzed with Logistic Regression										
	Sample Size, N		ACE, %		BB, %		CCB, %		Diuretics, %	
	Cases, N	Controls, N	Case	Control	Case	Control	Case	Control	Case	Control
FHS	132	660	41.1%	40.4%	36.0%	34.1%	31.2%	22.2%	29.8%	29.2%
HVH-1	1285	1005	39.4%	36.6%	36.2%	34.6%	22.3%	18.5%	34.9%	40.4%
HVH-2	381	682	42.0%	45.0%	42.8%	35.8%	16.0%	18.0%	39.7%	44.6%
PROSPER	406	2621	22.4%	20.1%	35.5%	33.6%	37.9%	30.1%	57.1%	57.0%
RS	241	241	35.7%	38.2%	53.1%	56.4%	27.0%	22.8%	29.5%	38.2%
Stage II										
	Cases, N		ACE, %		BB, %		CCB, %		Diuretics, %	
GenHAT	1751		27%		n/a		28%		47%	
AA Participants										
Analyzed with Cox Proportional Hazards Regression										
	Sample Size, N		ACE, %		BB, %		CCB, %		Diuretics, %	
ARIC	1059		24.0%		22.0%		23.0%		47.0%	
CHS	497		32.0%		20.0%		44.0%		50.0%	
Health ABC	707		46.0%		24.0%		45.0%		45.0%	
MESA	888		45.9%		26.0%		38.0%		56.8%	
GenHAT	888		30%		n/a		26%		48%	
Analyzed with Logistic Regression										
	Sample Size, N		ACE, %		BB, %		CCB, %		Diuretics, %	
	Cases, N	Controls, N	Case	Control	Case	Control	Case	Control	Case	Control
JHS	34	68	67.7%	11.8%	26.5%	51.5%	50.0%	33.8%	44.0%	52.8%

Table shows prevalence of antihypertensive use by study. ACE indicates Angiotensin-converting enzyme inhibitor; BB, beta-blocker; CCB, calcium channel blocker; Diuretics, thiazide diuretics.

Supplementary Table D: Number of SNPs and genomic inflation factors for discovery meta-analyses

Outcome	Drug	SNPs (N)	lambda
CVD	ACE inhibitors	1,892,626	0.9928
CVD	Beta blockers	1,971,049	0.9769
CVD	Calcium Channel Blockers	1,807,456	0.9985
CVD	Diuretics	2,030,893	0.9972
MI	ACE inhibitors	1,465,692	0.9816
MI	Beta blockers	1,780,479	0.9870
MI	Calcium Channel Blockers	1,194,322	0.9789
MI	Diuretics	1,822,485	0.9907

Table shows the number of SNPs available for analysis after QC parameters were applied at the level of the individual studies and across all studies contributing to the discovery meta-analysis. Lambda indicates the genomic inflation factor.

Supplementary Table E: Top associations for SNP×anti-hypertensive interactions on incident CVD risk from Stage I & Stage II meta-analysis

ACE				CHARGE Discovery Meta			GenHAT Extension		CHARGE + GenHAT Meta		
**SNPID**	**Chr:Position**	**gene**	**A1/2**	**AF**	**B**	**P**	**B**	**P**	**B**	**P**	**N**
rs4077408	1:208827067	*HHAT*	A/G	0.69	0.26	3.0E-04	0.23	7.9E-03	0.25	7.6E-06	15,276
rs1125311	4:55008230	*PDGFRA*	T/C	0.12	0.59	7.3E-05	0.24	3.2E-02	0.37	4.1E-05	5,925
rs12780491	10:96111012	*NOC3L*	A/G	0.76	-0.29	8.8E-05	-0.15	9.2E-02	-0.23	4.4E-05	15,486
rs6539804	12:82857661	*TMTC2*	A/G	0.41	-0.25	1.0E-04	-0.12	1.2E-01	-0.20	6.6E-05	16,523
rs6053888	20:6010330	*C20orf42*	T/G	0.83	0.36	3.4E-04	0.21	5.4E-02	0.29	8.4E-05	12,592
rs11857438	15:56262198	*AQP9*	T/C	0.27	-0.27	3.6E-04	-0.17	6.4E-02	-0.23	8.9E-05	15,307
rs10140322*	14:80665681	*TSHR*	C/G	0.38	0.27	1.7E-04	0.13	1.0E-01	0.21	9.7E-05	14,497
rs794733	5:173807592	*MSX2*	T/C	0.73	-0.34	7.4E-06	-0.07	4.1E-01	-0.22	1.0E-04	14,621
rs7311282*	12:83406153	*SLC6A15*	T/C	0.47	0.29	3.4E-04	0.15	5.3E-02	0.21	1.1E-04	13,207
rs2896481*	12:82831096	*TMTC2*	C/G	0.67	-0.25	3.9E-04	-0.15	7.7E-02	-0.21	1.1E-04	15,301
**CCB**				**CHARGE Discovery Meta**			**GenHAT Extension**		**CHARGE + GenHAT Meta**		
**SNPID**	**Chr:Position**	**gene**	**A1/2**	**AF**	**B**	**P**	**B**	**P**	**B**	**P**	**N**
rs3768939	2:236654796	*CENTG2*	A/G	0.19	-0.37	1.5E-04	-0.33	1.6E-03	-0.35	8.5E-07	11,378
rs7751419	6:39223391	*C6orf64*	T/C	0.29	0.30	1.5E-04	0.22	6.0E-03	0.26	3.4E-06	14,064
rs1397845	6:24118321	*NRSN1*	C/G	0.78	-0.46	3.6E-07	-0.06	5.1E-01	-0.26	5.0E-05	12,180
rs10513285	3:147852812	*PLSCR5*	C/G	0.15	0.54	1.7E-05	0.17	1.1E-01	0.33	5.6E-05	8,245
rs10848922	12:3731542	*EFCAB4B*	T/C	0.17	0.45	3.4E-04	0.23	2.3E-02	0.32	5.9E-05	7,943
rs1106262*	17:27102830	*C17orf79*	T/C	0.65	-0.24	5.3E-04	-0.17	3.4E-02	-0.21	6.3E-05	15,201
rs10747814	12:57249966	*LRIG3*	A/G	0.58	0.29	6.8E-05	0.12	1.1E-01	0.21	6.6E-05	13,903
rs6103672	20:42255253	*C20orf111*	T/C	0.19	-0.45	2.5E-06	-0.09	3.9E-01	-0.28	6.6E-05	11,440
rs12186728	5:53973230	*SNAG1*	T/C	0.15	0.60	6.7E-05	0.21	5.0E-02	0.34	9.2E-05	6,558
rs2298765	11:69017173	*CCND1*	T/C	0.17	-0.45	4.6E-05	-0.16	1.5E-01	-0.30	1.0E-04	10,041
**Diuretics**				**CHARGE Discovery Meta**			**GenHAT Extension**		**CHARGE + GenHAT Meta**		
**SNPID**	**Chr:Position**	**gene**	**A1/2**	**AF**	**B**	**P**	**B**	**P**	**B**	**P**	**N**
rs10514740	3:80552658	*ROBO1*	T/C	0.06	-0.74	4.1E-05	-0.34	2.5E-02	-0.51	1.3E-05	8,352
rs2331493	14:21638312	*OR4E2*	C/G	0.84	0.35	1.0E-04	0.19	4.1E-02	0.27	2.6E-05	13,416
rs4813344	20:18778812	*C20orf79*	T/C	0.34	-0.29	2.1E-04	-0.16	3.0E-02	-0.22	3.8E-05	12,754
rs6854402	4:66812215	*EPHA5*	T/C	0.75	0.29	2.2E-04	0.16	4.0E-02	0.23	4.8E-05	13,853
rs686652	9:134845393	*GFI1B*	T/C	0.13	-0.39	3.1E-04	-0.22	3.3E-02	-0.30	5.5E-05	10,324
rs4822842	22:25801415	*CRYBA4*	T/C	0.92	-0.61	6.3E-05	-0.23	7.4E-02	-0.39	7.9E-05	8,335
rs2619681	15:38776313	*RAD51*	T/C	0.16	0.35	2.0E-04	0.17	6.2E-02	0.26	8.1E-05	13,203
rs4964537	12:106151146	*BTBD11*	T/C	0.38	-0.25	1.0E-04	-0.11	1.2E-01	-0.19	8.9E-05	16,103
rs17694459	18:13735055	*RNMT*	A/G	0.22	-0.34	3.2E-04	-0.16	5.5E-02	-0.24	1.4E-04	11,432
rs11238609	10:43453997	*ZNF32*	T/C	0.30	0.32	2.0E-06	0.03	6.8E-01	0.19	1.5E-04	15,851

* indicates proxy SNP; Chr:Position, hg18 chromosome & position; gene, the nearest gene; A1/2, coded & non-coded allele; AF, coded allele frequency; B, beta for SNPxdrug interaction term; P, p-value for interaction; N, effective sample size.

When multiple SNPs from a single locus clustered among the top associations, the SNP with the smallest meta-analysis p-value was retained and other secondary SNPs with $r^2 > 0.6$ based on the HapMap Phase II CEU reference panel were removed from table

Supplementary Table F: Top associations for SNP×anti-hypertensive interactions on incident CVD risk from Stage I & Stage II meta-analysis among African-American Participants

ACE		European Ancestry Meta (Stage I + II)					African-American Meta				
**SNP**	**Gene**	**A1/2**	**AF**	**B**	**P**	**N**	**A1/2**	**AF**	**B**	**P**	**N**
rs4077408	*HHAT*	A/G	0.69	0.25	7.6E-06	15,276	A/G	0.88	0.09	0.50	3,114
rs1125311	*PDGFRA*	T/C	0.12	0.37	4.1E-05	5,925	T/C	0.04	0.05	0.86	2,109
rs12780491	*NOC3L*	A/G	0.76	-0.23	4.4E-05	15,486	A/G	0.75	-0.05	0.66	3,295
rs6539804	*TMTC2*	A/G	0.41	-0.20	6.6E-05	16,523	A/G	0.24	-0.02	0.84	3,202
rs6053888	*C20orf42*	T/G	0.83	0.29	8.4E-05	12,592	T/G	0.64	0.11	0.28	2,613
rs11857438	*AQP9*	T/C	0.27	-0.23	8.9E-05	15,307	T/C	0.25	0.18	0.07	3,257
rs10140322*	*TSHR*	C/G	0.38	0.21	9.7E-05	14,497	C/G	0.77	-0.06	0.59	3,136
rs794733	*MSX2*	T/C	0.73	-0.22	1.0E-04	14,621	T/C	0.52	-0.06	0.50	3,259
rs7311282*	*SLC6A15*	T/C	0.47	0.21	1.1E-04	13,207	T/C	0.47	0.02	0.84	1,630
rs2896481*	*TMTC2*	C/G	0.67	-0.21	1.1E-04	15,301	C/G	0.78	-0.02	0.89	1,608
**CCB**		**European Ancestry Meta (Stage I + II)**					**African-American Meta**				
**SNP**	**Gene**	**A1/2**	**AF**	**B**	**P**	**N**	**A1/2**	**AF**	**B**	**P**	**N**
rs3768939	*CENTG2*	A/G	0.19	-0.35	8.5E-07	11,378	A/G	0.35	0.08	0.43	3,292
rs7751419	*C6orf64*	T/C	0.29	0.26	3.4E-06	14,064	T/C	0.17	0.01	0.93	3,188
rs1397845	*NRSN1*	C/G	0.78	-0.26	5.0E-05	12,180	C/G	0.59	-0.09	0.31	3,127
rs10513285	*PLSCR5*	C/G	0.15	0.33	5.6E-05	8,245	C/G	0.05	-0.03	0.89	2,156
rs10848922	*EFCAB4B*	T/C	0.17	0.32	5.9E-05	7,943	T/C	0.21	-0.27	0.02	3,262
rs1106262*	*C17orf79*	T/C	0.65	-0.21	6.3E-05	15,201	T/C	0.72	-0.03	0.75	3,206
rs10747814	*LRIG3*	A/G	0.58	0.21	6.6E-05	13,903	A/G	0.39	0.04	0.66	3,274
rs6103672	*C20orf111*	T/C	0.19	-0.28	6.6E-05	11,440	T/C	0.08	-0.35	0.06	1,578
rs12186728	*SNAG1*	T/C	0.15	0.34	9.2E-05	6,558	T/C	0.24	0.10	0.35	2,621
rs2298765	*CCND1*	T/C	0.17	-0.30	1.0E-04	10,041	T/C	0.13	0.02	0.86	2,662
**Diuretics**		**European Ancestry Meta (Stage I + II)**					**African-American Meta**				
**SNP**	**Gene**	**A1/2**	**AF**	**B**	**P**	**N**	**A1/2**	**AF**	**B**	**P**	**N**
rs10514740	*ROBO1*	T/C	0.06	-0.51	1.3E-05	8,352	T/C	0.11	-0.35	0.02	3,109
rs2331493	*OR4E2*	C/G	0.84	0.27	2.6E-05	13,416	C/G	0.88	-0.15	0.26	1,864
rs4813344	*C20orf79*	T/C	0.34	-0.22	3.8E-05	12,754	T/C	0.28	0.00	1.00	1,989
rs6854402	*EPHA5*	T/C	0.75	0.23	4.8E-05	13,853	T/C	0.34	0.09	0.31	2,925
rs686652	*GFI1B*	T/C	0.13	-0.30	5.5E-05	10,324	T/C	0.16	-0.11	0.35	1,742
rs4822842	*CRYBA4*	T/C	0.92	-0.39	7.9E-05	8,335	T/C	0.73	0.08	0.46	2,406
rs2619681	*RAD51*	T/C	0.16	0.26	8.1E-05	13,203	T/C	0.26	0.00	0.98	462
rs4964537	*BTBD11*	T/C	0.38	-0.19	8.9E-05	16,103	T/C	0.34	-0.09	0.33	3,062
rs17694459	*RNMT*	A/G	0.22	-0.24	1.4E-04	11,432	A/G	0.05	0.42	0.05	1,988
rs11238609	*ZNF32*	T/C	0.30	0.19	1.5E-04	15,851	T/C	0.36	0.09	0.33	3,172

* indicates proxy SNP; Chr:Position, hg18 chromosome & position; gene, the nearest gene; A1/2, coded & non-coded allele; AF, coded allele frequency; B, beta for SNPxdrug interaction term; P, p-value for interaction; N, effective sample size.

When multiple SNPs from a single locus clustered among the top associations, the SNP with the smallest meta-analysis p-value was retained and other secondary SNPs with $r^2 > 0.6$ based on the HapMap Phase II CEU reference panel were removed from table

Supplementary Table G: Top associations for SNP×anti-hypertensive interactions on incident MI risk from Stage I & Stage II meta-analysis

**ACE**				**CHARGE Discovery Meta**			**GenHAT Extension**		**CHARGE + GenHAT Meta**		
**SNPID**		**gene**	**A1/2**	**AF**	**B**	**P**	**B**	**P**	**B**	**P**	**N**
rs9344943	6:90303456	*LYRM2*	T/C	0.56	-0.34	6.3E-05	-0.16	8.3E-02	-0.26	3.6E-05	12,243
rs6793321	3:160948793	*SCHIP1*	T/C	0.26	0.44	2.5E-05	0.15	1.5E-01	0.30	6.0E-05	10,764
rs1340129	10:16354723	*PTER*	A/C	0.18	-0.64	5.7E-05	-0.24	7.1E-02	-0.40	7.7E-05	5,989
rs6544620	2:43038311	*HAAO*	A/G	0.56	0.31	2.3E-04	0.17	8.3E-02	0.25	8.6E-05	13,462
rs4349629	4:63640765	*LPHN3*	T/C	0.30	0.39	4.2E-04	0.21	4.3E-02	0.30	1.0E-04	9,802
rs10938694	4:8222301	*ABLIM2*	T/C	0.42	0.33	2.0E-04	0.16	9.1E-02	0.25	1.1E-04	11,999
rs4725649	7:143734050	*NOBOX*	T/C	0.41	0.34	8.8E-05	0.14	1.6E-01	0.25	1.2E-04	12,432
rs1527033	8:24945829	*NEFL*	T/C	0.39	-0.34	1.6E-04	-0.16	1.2E-01	-0.26	1.2E-04	11,395
rs1797994	12:39795888	*CNTN1*	A/C	0.46	0.36	3.9E-05	0.11	2.6E-01	0.25	1.3E-04	11,895
rs1472962	4:95811400	*PDLIM5*	A/G	0.59	-0.38	1.3E-05	-0.08	4.3E-01	-0.24	1.6E-04	12,338
**CCB**				**CHARGE Discovery Meta**			**GenHAT Extension**		**CHARGE + GenHAT Meta**		
**SNPID**		**gene**	**A1/2**	**AF**	**B**	**P**	**B**	**P**	**B**	**P**	**N**
rs16964543	19:35877050	*ZNF536*	T/C	0.67	-0.46	1.4E-05	-0.29	3.6E-03	-0.37	3.6E-07	10,447
rs827783*	5:169767143	*KCNMB1*	A/G	0.68	0.39	3.3E-04	0.27	1.0E-02	0.32	1.5E-05	10,376
rs6432267*	2:12235917	*LPIN1*	A/G	0.50	-0.33	3.7E-04	-0.21	2.6E-02	-0.27	4.1E-05	11,990
rs11800010	1:236774778	*ZP4*	A/T	0.60	0.38	3.7E-04	0.20	4.0E-02	0.28	8.9E-05	9,428
rs11134628*	5:169802917	*KCNMB1*	A/G	0.24	-0.54	2.2E-05	-0.17	1.4E-01	-0.33	9.4E-05	7,845
rs17197854	4:102826806	*BANK1*	T/C	0.72	0.72	1.3E-08	0.03	7.5E-01	0.32	9.9E-05	9,174
rs10765041*	10:132835751	*TCERG1L*	T/G	0.34	-0.40	4.5E-04	-0.21	3.7E-02	-0.29	1.0E-04	9,952
rs1322489	1:167227418	*ATP1B1*	A/C	0.55	0.34	6.6E-04	0.19	5.5E-02	0.26	1.7E-04	11,237
rs10745808	12:96723813	*TMPO*	A/C	0.29	0.55	2.0E-05	0.14	1.7E-01	0.30	1.9E-04	7,989
rs9858039*	3:74207093	*CNTN3*	T/C	0.52	0.33	4.1E-04	0.16	9.4E-02	0.24	2.3E-04	12,017
**Diuretics**				**CHARGE Discovery Meta**			**GenHAT Extension**		**CHARGE + GenHAT Meta**		
**SNPID**		**gene**	**A1/2**	**AF**	**B**	**P**	**B**	**P**	**B**	**P**	**N**
rs7930533	11:27599900	*BDNF*	A/G	0.45	0.40	2.1E-05	0.18	3.8E-02	0.28	1.1E-05	10,093
rs9318282	13:73980589	*KLF12*	T/C	0.72	-0.35	1.8E-04	-0.20	3.0E-02	-0.28	3.0E-05	11,793
rs6552734	4:185189917	*STOX2*	T/C	0.68	-0.51	1.6E-05	-0.17	5.3E-02	-0.30	3.3E-05	8,309
rs4272042	4:78272999	*CCNG2*	A/G	0.70	0.46	3.3E-04	0.23	1.3E-02	0.31	3.8E-05	7,674
rs6845751	4:76184547	*DKFZP564O0823*	T/G	0.19	0.41	3.1E-04	0.24	2.4E-02	0.32	3.9E-05	10,246
rs1415687	9:105259104	*CYLC2*	A/G	0.26	-0.34	3.9E-04	-0.22	2.8E-02	-0.28	4.4E-05	11,496
rs2479561	13:26497574	*USP12*	T/C	0.39	0.32	2.3E-04	0.18	4.9E-02	0.25	5.9E-05	11,935
rs10759186	9:108355131	*ZNF462*	T/C	0.33	-0.39	2.2E-05	-0.13	1.6E-01	-0.26	6.9E-05	11,527
rs17832899	18:70653700	*ZNF407*	A/G	0.75	0.42	9.7E-05	0.18	7.4E-02	0.29	8.0E-05	9,825
rs7300022	12:37854158	*KIF21A*	A/G	0.37	0.34	1.5E-04	0.15	8.7E-02	0.24	1.1E-04	11,784

* indicates proxy SNP; Chr:Position, hg18 chromosome & position; gene, the nearest gene; A1/2, coded & non-coded allele; AF, coded allele frequency; B, beta for SNPxdrug interaction term; P, p-value for interaction; N, effective sample size.

When multiple SNPs from a single locus clustered among the top associations, the SNP with the smallest meta-analysis p-value was retained and other secondary SNPs with $r^2 > 0.6$ based on the HapMap Phase II CEU reference panel were removed from table

Supplementary Table H: Top associations for SNP×beta-blocker interactions on incident CVD risk from Stage I meta-analysis

				CHARGE Discovery Meta		
**SNP**	**Chr:Position**	**gene**	**A1/2**	**AF**	**B**	**P**
rs973862	11:29212709	*KCNA4*	T/C	0.34	0.36	3.5E-06
rs8097092	18:406138	*COLEC12*	T/C	0.37	0.34	9.1E-06
rs6656669	1:208635858	*HHAT*	A/G	0.83	0.44	9.5E-06
rs2277115	6:47117910	*GPR110*	C/G	0.41	0.38	1.1E-05
rs6934968	6:23703118	*NRSN1*	A/G	0.21	-0.40	1.2E-05
rs4981802	14:19720867	*OR11G2*	T/C	0.23	0.40	1.4E-05
rs6704691	2:172167210	*CYBRD1*	C/G	0.78	0.39	1.4E-05
rs11030635	11:29214987	*KCNA4*	A/C	0.29	0.36	1.6E-05
rs1518793	2:14953809	*FAM84A*	T/C	0.88	0.48	2.0E-05
rs6474915	9:15405461	*SNAPC3*	A/G	0.31	-0.30	2.1E-05

* indicates proxy SNP; Chr:Position, hg18 chromosome & position; gene, the nearest gene; A1/2, coded & non-coded allele; AF, coded allele frequency; B, beta for SNPxdrug interaction term; P, p-value for interaction; N, effective sample size.

When multiple SNPs from a single locus clustered among the top associations, the SNP with the smallest meta-analysis p-value was retained and other secondary SNPs with r^2 > 0.6 based on the HapMap Phase II CEU reference panel were removed from table

Supplementary Table I: Interaction of Coronary Artery Disease GWAS SNP×antihypertensive use interactions on incident CVD risk from Stage I meta-analysis

SNP	Chr:Pos	Gene	ACE (P)	BB (P)	CCB (P)	Diuretics (P)
rs11206510	1:55268627	*PCSK9*	0.26	0.92	0.01	0.75
rs602633	1:109623034	*PSRC1*	0.82	0.58	0.97	0.14
rs4845625	1:152688691	*IL6R*	0.91	0.67	0.62	0.46
rs515135	2:21139562	*APOB*	0.18	0.25	0.34	0.87
rs1561198	2:85663500	*VAMP8*	0.79	0.99	0.74	0.96
rs2252641	2:145517931	*ZEB2*	0.44	0.71	0.56	0.21
rs6725887	2:203454130	*WDR12*	0.14	0.20	0.30	0.61
rs9818870	3:139604812	*MRAS*	0.73	0.26	0.86	0.56
rs7692387	4:156854759	*GUCY1A3*	0.68	0.77	0.85	0.34
rs9369640	6:13009427	*PHACTR1*	0.61	0.47	0.21	0.46
rs12205331	6:35006433	*ANKS1A*	0.68	0.20	0.03	0.06
rs10947789	6:39282900	*KCNK5*	0.85	0.21	0.10	0.98
rs12190287	6:134256218	*TCF21*	0.36	0.89	0.73	0.67
rs2048327	6:160783522	*SLC22A3*	0.47	0.33	0.70	0.05
rs4252120	6:161063598	*PLG*	0.44	0.76	0.50	0.33
rs12539895	7:106879085	*GPR22*	0.91	0.73	0.79	0.49
rs11556924	7:129450732	*ZC3HC1*	0.92	0.18	0.04	0.72
rs264	8:19857460	*LPL*	0.80	0.49	0.28	0.96
rs3217992	9:21993223	*CDKN2B*	0.58	0.45	0.61	0.59
rs1333049	9:22115503	*CDKN2B*	0.34	0.28	0.64	0.02
rs579459	9:135143989	*ABO*	0.76	0.43	0.61	0.84
rs2505083	10:30375128	*KIAA1462*	0.79	0.45	0.89	0.42
rs2047009	10:43859919	*CXCL12*	0.19	0.31	0.51	0.49
rs501120	10:44073873	*CXCL12*	0.79	0.21	0.24	0.08
rs11203042	10:90979089	*LIPA*	0.65	0.80	0.30	0.54
rs2246833	10:90995834	*LIPA*	0.85	0.67	0.22	0.89
rs974819	11:103165777	*PDGFD*	0.04	0.62	0.06	0.19
rs3184504	12:110368991	*SH2B3*	0.81	0.33	0.06	0.04
rs9319428	13:27871621	*FLT1*	0.26	0.52	0.22	0.41
rs4773144	13:109758713	*COL4A2*	0.83	0.43	0.76	0.17
rs9515203	13:109847624	*COL4A2*	0.35	0.84	0.15	0.40
rs2895811	14:99203695	*KIAA1822*	0.45	0.73	0.09	0.41
rs7173743	15:76928839	*MORF4L1*	0.21	0.36	0.77	0.22
rs17514846	15:89217554	*FURIN*	0.47	0.52	0.38	0.13
rs2281727	17:2064695	*SMG6*	0.70	0.24	0.79	0.88
rs12936587	17:17484447	*RAI1*	0.20	0.38	0.20	0.003
rs15563	17:44360192	*UBE2Z*	0.39	0.20	0.88	0.39
rs1122608	19:11024601	*SMARCA4*	0.97	0.59	0.47	0.73
rs2075650	19:50087459	*TOMM40*	0.97	0.20	0.73	0.62
rs9982601	21:34520998	*MRPS6*	0.91	0.05	0.89	0.71

Numbers in table are P-values for interaction from analyses of SNP×drug interaction analyses: ACE indicates Angiotensin-converting enzyme inhibitor; BB, beta-blocker; CCB, calcium channel blocker; Diuretics, thiazide diuretics; Chr:Position, indicates, hg18 chromosome & position; gene is the nearest gene.

Supplementary Table J: Interaction of Blood Pressure GWAS SNP×antihypertensive use interactions on incident CVD risk from Stage I meta-analysis

SNP	Chr:Pos	ClosestRefGene	ACE (P)	BB (P)	CCB (P)	Diuretics (P)
rs17367504	1:11785365	*MTHFR*	0.12	0.13	0.82	0.18
rs2932538	1:113018066	*MOV10*	0.91	0.73	0.72	0.35
rs13082711	3:27512913	*SLC4A7*	0.66	0.74	0.57	0.92
rs3774372	3:41852418	*ULK4*	0.22	0.83	0.86	0.55
rs419076	3:170583580	*MDS1*	0.49	0.89	0.82	0.13
rs1458038	4:81383747	*FGF5*	0.70	0.77	0.17	0.69
rs13139571	4:156864963	*GUCY1A3*	0.25	0.25	0.92	0.19
rs1173771	5:32850785	*C5orf23*	0.52	0.59	0.01	0.98
rs11953630	5:157777980	*EBF1*	0.43	0.39	0.03	0.57
rs1799945	6:26199158	*HFE*	0.02	0.60	0.79	0.37
rs805303	6:31724345	*BAT3*	0.04	0.06	0.97	0.30
rs4373814	10:18459978	*CACNB2*	0.59	0.44	0.25	0.34
rs1813353	10:18747454	*CACNB2*	0.80	0.90	0.79	0.79
rs4590817	10:63137559	*C10orf107*	0.73	0.06	0.01	0.50
rs932764	10:95885930	*PLCE1*	0.32	0.60	0.62	0.78
rs7129220	11:10307114	*ADM*	0.22	0.48	0.05	0.37
rs381815	11:16858844	*PLEKHA7*	0.34	0.68	0.73	0.34
rs633185	11:100098748	*TMEM133*	0.27	0.66	0.21	0.07
rs17249754	12:88584717	*ATP2B1*	0.12	0.26	0.78	0.85
rs3184504	12:110368991	*SH2B3*	0.81	0.33	0.06	0.04
rs10850411	12:113872179	*TBX3*	0.27	0.27	0.02	0.27
rs1378942	15:72864420	*CSK*	0.94	0.54	0.08	0.62
rs2521501	15:89238392	*FES*	0.13	0.04	0.78	0.47
rs12940887	17:44757806	*ZNF652*	0.09	0.95	0.94	0.60
rs1327235	20:10917030	*JAG1*	0.19	0.74	0.81	0.05
rs6015450	20:57184512	*C20orf174*	0.76	0.54	0.27	0.05

Numbers in table are P-values for interaction from analyses of SNP×drug interaction analyses: ACE indicates Angiotensin-converting enzyme inhibitor; BB, beta-blocker; CCB, calcium channel blocker; Diuretics, thiazide diuretics; Chr:Position, indicates, hg18 chromosome & position; gene is the nearest gene.

Section A: Study Descriptions

Age, Gene, Environment, Susceptibility—Reykjavik Study (AGES)

The Reykjavik Study cohort originally comprised a random sample of 30,795 men and women born in 1907–1935 and living in Reykjavik in 1967. A total of 19,381 attended, resulting in 71% recruitment rate. Between 2002 and 2006, the AGES-Reykjavik study re-examined 5,764 survivors of the original cohort who had participated before in the Reykjavik Study [1]. Information on CHD events was taken from hospital records, and those with prevalent CHD were excluded from the analyses.

Atherosclerosis Risk in Communities (ARIC) Study

The Atherosclerosis Risk in Communities (ARIC) study is a prospective study of 15,792 men and women aged 45–64 years at baseline (1987–1989) [2].The cohort was sampled from four United States communities (Forsyth County, NC; Jackson, MS; suburban Minneapolis, MN; Washington County, MD) and followed prospectively until incident coronary heart disease (CHD) or stroke, loss to follow-up, death, or December 31, 2008.

Cardiovascular Health Study (CHS)

The Cardiovascular Health Study (CHS) is a population-based cohort study of risk factors for CHD and stroke in adults ≥65 years conducted across four field centers [3]. The original predominantly Caucasian cohort of 5,201 persons was recruited in 1989–1990 from random samples of the Medicare eligibility lists; subsequently, an additional predominantly African-American cohort of 687 persons were enrolled for a total sample of 5,888. Blood samples were drawn on all participants at their baseline examination and DNA was subsequently extracted. CHS was approved by institutional review committees at each site, the subjects gave informed consent, and those included in the present analysis consented to the use of their genetic information for the study of cardiovascular disease.

Genotyping was performed at the General Clinical Research Center's Phenotyping/Genotyping Laboratory at Cedars-Sinai in two phases: In 2007, 3,980 CHS participants who were free of CVD at baseline, consented to genetic testing, and had DNA available for genotyping were genotyped using the Illumina 370CNV BeadChip system; subsequently, in 2010, 844 African-American CHS participants who

consented to genetic testing, and had DNA available for genotyping were genotyped using the Illumina HumanOmni1-Quad_v1 BeadChip system.

For these analyses, the study sample was limited to participants with pharmaceutically-treated hypertension who were free of CVD at baseline and had available genotype and follow-up data.

Framingham Heart Study (FHS):

The Framingham Heart Study started in 1948 with 5,209 ascertained adult participants from two-thirds of the households of Framingham, Massachusetts, United States, who have undergone biannual examinations to investigate cardiovascular diseases and its risk factors [4, 5]. In 1971, the Offspring Cohort [6, 7] (comprising 5,124 children of the original cohort and children's spouses) and in 2002, the Third Generation (consisting of 4,095 children of the Offspring Cohort) were recruited.[8]

The current study was conducted in 792 participants of the Original and Offspring cohorts who were free of CVD at the time of their blood draw for a DNA sample. Due to DNA quality considerations, we only include subjects who had DNA drawn between 1985 and 2003 in the Original Cohort or after 1987 in the Offspring Cohort. All subjects were between 30 and 79 years of age at baseline and received treatment for hypertension, with one of following four drugs: diuretics (thiazide or potassium-sparking, but not loop); beta-blockers; calcium channel blockers; angiotensin-converting enzyme inhibitors (including angiotensin receptor blockers).

Of these, 162 developed CVD after their DNA draw. An Endpoint Committee consisting of three physicians reviewed all suspected cardiovascular disease events to confirm occurrence of the event, using previously agreed upon criteria. MI in this project was defined as a recognized non-fatal MI diagnosed with documented evidence from EKG or elevated enzymes; CVD was defined as recognized MI, stroke (excluding Transient Ischemic Attacks), congestive heart failure or death due to CVD. For each incident case, five subjects who were free of MI, CHF, stroke and still alive after the case's event date were chosen from the same cohort, same gender, and within a decade of age as the case's.

The Framingham Heart Study was approved by the institutional review boards of Boston University and the National Institutes of Health. All participants provided written informed consent.

Health, Aging, and Body Composition Study (Health ABC)

The Health, Aging, and Body Composition (Health ABC) Study is a NIA-sponsored cohort study of the factors that contribute to incident disability and the decline in function of healthier older persons, with a particular emphasis on changes in body composition in old age. Between 15 April 1997 and 5 June 1998, the Health ABC study recruited 3,075 70–79-year-old community-dwelling adults (41% African-American), who were initially free of mobility and activities of daily living disability. The key components of Health ABC include a baseline exam, annual follow-up clinical exams, and phone contacts every 6 months to identify major health events and document functional status between clinic visits. Provision has been made for banking of blood specimens and extracted DNA.

Heart and Vascular Health Study (HVH)

The setting for Heart and Vascular Health Study (HVH) was Group Health (GH), a large integrated health care system in western Washington State. Data were utilized from a series of ongoing case-control studies of incident myocardial infarction (MI), stroke cases, and sudden cardiac arrest cases with a shared common control group. Methods for the study have been described previously and are briefly summarized below [9-12]. The study was approved by the human subjects committee at GH, and written informed consent was provided by all study participants.

All study participants were GH members and aged 30–79 years. MI and stroke cases were identified from hospital discharge diagnosis codes and were validated by medical record review. Sudden cardiac arrest cases were GH enrollees attended by paramedics in Seattle and King Country between 1988 and 2004. Controls were a random sample of GH members frequency matched to MI cases on age (within decade), sex, treated hypertension, and calendar year of identification. The index date for controls was a computer-generated random date within the calendar year for which they had been selected. For MI cases, the index date was the date of admission for the first acute MI. Participants were excluded if they were recent enrollees at GH, had a history of prior MI, stroke, or cardiac arrest, or if the incident event was a complication of a procedure or surgery.

Eligibility and risk factor information were collected by trained medical record abstractors from a review of the GH medical record using only data available prior to the index date and through a telephone interview. Medication use was ascertained using computerized GH pharmacy records. A venous blood sample was collected from all consenting subjects, and DNA was extracted from white blood cells using

standard procedures. For sudden cardiac arrest cases, blood samples were collected by paramedics at the time of the cardiac arrest.

Jackson Heart Study (JHS)

The Jackson Heart Study (JHS) is a prospective population-based study established in 2000 to study the causes of greater burden of cardiovascular diseases among African Americans [13-15]. Four strategies were employed to recruit a total of 5,301 participants in the first examination cycle (2000 to 2004) from Jackson, Mississippi metropolitan tri-counties of Hinds, Madison and Rankin[13]; (i) all living Jackson, Mississippi participants enrolled in the Atherosclerosis Risk in Communities (ARIC) study aged 35–84 years at initiation of the study (31%) were invited to participate, also referred to as the ARIC–JHS overlap; (ii) a random sample (17%) recruited from a commercially available list (AccuData Integrated Marketing, Fort Myers, Florida); (iii) a volunteer sample (30%) aged between 35–84 years who responded to targeted advertisements: radio, newspaper, local churches, and civic/social organizations; and (iv) family members (22%) mainly family members of enumerated households including those at least 21 old for purposes of genetic studies. Participants recruited in strategies ii-iv are hereby referred to as the JHS-specific sample. Of the total recruited participants, 3,028 or 57.1 percent (892 ARIC–JHS overlap and 2,136 JHS-specific) gave consent for genetic analyses and thus were genotyped in the CARe consortium[16] using Affymetrix 6.0 platform.

For this study we excluded participants in the ARIC–JHS overlap.

Multi-Ethnic Study of Atherosclerosis (MESA)

MESA is a study of the characteristics of subclinical cardiovascular disease (disease detected non-invasively before it has produced clinical signs and symptoms) and the risk factors that predict progression to clinically overt cardiovascular disease or progression of the subclinical disease [17]. MESA researchers study a diverse, population-based sample of 6,814 asymptomatic men and women aged 45–84 years. Thirty-eight percent of the recruited participants are white, 28 percent African-American, 22 percent Hispanic, and 12 percent Asian, predominantly of Chinese descent. Participants were recruited from six field centers across the United States. Four physical examinations (at baseline and at three follow-up time points) were selected for analysis. The tenets of the Declaration of Helsinki were followed and institutional review board approval was granted at all MESA sites. Written informed consent was obtained from each participant.

PROspective Study of Pravastatin in the Elderly at Risk (PROSPER)

All data come from the PROspective Study of Pravastatin in the Elderly at Risk (PROSPER). A detailed description of the study has been published elsewhere [18]. PROSPER was a prospective multicenter randomized placebo-controlled trial to assess whether treatment with pravastatin diminishes the risk of major vascular events in elderly [19]. Between December 1997 and May 1999, we screened and enrolled subjects in Scotland (Glasgow), Ireland (Cork), and the Netherlands (Leiden). Men and women aged 70–82 years were recruited if they had pre-existing vascular disease or increased risk of such disease because of smoking, hypertension, or diabetes. A total number of 5,804 subjects were randomly assigned to pravastatin or placebo. A large number of prospective tests were performed including Biobank tests and cognitive function measurements. A whole genome wide screening has been performed in the sequential PHASE project with the use of the Illumina 660K beadchip [20]. Of 5,763 subjects for whom DNA was available for genotyping, after QC (call rate $<95\%$) 5,244 subjects and 557,192 SNPs were left for analysis. These SNPs were imputed to 2.5 million SNPs based on the HapMap build 36 with MaCH imputation software.

Rotterdam Study

The Rotterdam Study (RS-I) is a community-based study of elderly individuals from a suburb of Rotterdam with a focus on identifying determinants of health and cardiovascular, neurogeriatric, bone, and eye diseases [21, 22].

Participants aged ≥55 years were examined up to four times every three years. Participants were all 55 years of age or over when the study started in 1990. They were interviewed at home (2 hours) and then had an extensive set of examinations (a total of 5 hours) in a specially built research facility in the centre of their district. The emphasis was put on imaging (of heart, blood vessels, eyes, skeleton, and later, brain) and on collecting body fluids that enabled further in-depth molecular and genetic analyses. These examinations were repeated every 3–4 years in characteristics that could change over time. The participants in the Rotterdam Study are followed for a variety of diseases that are frequent in the elderly, such as coronary heart disease, heart failure, stroke, and dementia. The Rotterdam Study has been approved by the institutional review board (Medical Ethics Committee) of the Erasmus Medical Center and by the review board of The Netherlands Ministry of Health, Welfare and Sports.

Methods of data collection and adjudication of events have been described previously [22]. In short, Rotterdam Study participants are continuously followed-up by direct digital linkage of the study base with medical files from the collaborating general practitioners. Subsequently two independent research physicians and either an experienced cardiologist or neurologist checked each potential event. Myocardial infarction was defined as a reported myocardial infarction with hospital admission or the presence of a myocardial infarction on the ECG. More details on methods of follow up and cardiovascular disease event adjudication have been described previously [22].

Complete information on prescriptions for all participants was obtained in automated format from collaborating pharmacies.

Genetics of Hypertension Associated Treatment (GenHAT) study

The participants of this study were part of the Genetics of Hypertension Associated Treatment (GenHAT) study, an ancillary study of ALLHAT. The study design and methodology of GenHAT and ALLHAT have been previously described in detail [23, 24]. Briefly, ALLHAT was a randomized, double-blind, multicenter (623 sites) clinical trial of 42,418 hypertensive adults aged 55 years and older designed to determine if the incidence of fatal CHD and nonfatal myocardial infarction was lower among patients randomized to one of four antihypertensive drug classes: a calcium channel blocker (amlodipine), an angiotensin-converting enzyme inhibitor (lisinopril), and an alpha-adrenergic blocker (doxazosin), each compared with a diuretic (chlorthalidone), in an assignment ratio of 1:1:1:1.7, respectively. Treatment was given once daily and was titrated to achieve BP of 140/90 mmHg—chlorthalidone (12.5 mg for the first and second titration and 25 mg for the third), lisinopril (10, 20, or 40 mg), amlodipine (2.5, 5, or 10 mg), or doxazosin (2, 4, or 8 mg). If BP control was not achieved on the maximum study medication dose, a second-step, open-label agent (reserpine, clonidine, or atenolol) and/or a third-step open-label agent (hydralazine) was added. Information on study drug continuation, study drug cross-over, add-on antihypertensive therapy was recorded at yearly visits. Due to early termination of the doxazosin arm owing to futility for the primary outcome and a significant increase in the secondary outcome of CVD compared with the chlorthalidone arm, follow-up continued for an average of 3.2 years for the doxazosin arm[25] and 4.9 years for all other treatment arms.

The GenHAT study was designed to determine whether variants in HTN susceptibility genes interact with antihypertensive medication to modify coronary heart disease (CHD) risk in hypertensives [24]. In the case-only phase of GenHAT, 11,599 ALLHAT participants who experienced an adverse event

(fatal CHD or nonfatal myocardial infarction, stroke, HF, coronary revascularization, angina, peripheral arterial disease, end-stage renal disease, all-cause death), were successfully genotyped for about 600 polymorphisms in genes selected for their associations with BP regulation and CVD, with the goal of discovering pharmacogenetic associations with the genes [26]. This study focuses on two possible outcomes: the CHD (primary ALLHAT outcome) or stroke (N=2639 with genotype data). Outcomes were reported by clinical investigators, and documentation (death certificate and hospital discharge summary) was submitted for any outcome involving death or hospitalization. National databases (Center for Medicare and Medicaid Services, the Department of Veteran's Affairs, the National Death Index, and the Social Security Administration) were also used to identify deaths occurring among participants lost to follow-up. A complete description of outcome ascertainment has been previously published [23, 25, 27].

The documentation of CVD outcomes in ALLHAT has been described in detail elsewhere [25]. In brief, because ALLHAT was a large, simple trial, study outcomes were counted based on clinic investigator report and reported to the clinical trials center (CTC). For deaths and hospitalizations, copies of death certificates and hospital discharge summaries were requested. These documents were used to support the clinician-assigned diagnoses. All documents were verified for completion and appropriateness by the CTC. For a random (10%) subset of events, more detailed information (e.g. enzyme levels for CHD and computed tomography and/or magnetic resonance imaging reports for strokes) was requested by the CTC and reviewed by the Endpoints Subcommittee to validate the procedure of using clinician diagnoses with accompanying documentation. The agreement rate was 92% (72/78) for the primary end point [25].

Section B: Methods for outcome and exposure ascertainment

Age, Gene, Environment, Susceptibility—Reykjavik Study (AGES):

Methods for phenotype ascertainment for the AGES study has been described elsewhere [1]; a summary is provided below.

Exclusions	n/a
CVD definition	Recorded cause of death. When listed as CHD
MI definition	Hospital codes used, ICD-9: 410* and ICD-10: I21*,I22*
Surveillance Method	Hospital Records
Medication ascertainment methods:	Participants brought with them all medication they were taking

Atherosclerosis Risk in Communities (ARIC) Study:

Methods for phenotype ascertainment for the ARIC study has been described elsewhere [28-31] ; a summary is provided below.

Exclusions	Baseline history of myocardial infarction, stroke, heart failure (time-dependent).<br>No history of hypertension<br>Untreated with Thiazide, beta-blocker, calcium channel blocker, ACE inhibitor, or angtiotensin-receptor blocker.
CVD definition	Definite or probable myocardial infarction, fatal coronary heart disease, silent myocardial infarction, definite or probable stroke Incident CHD events included hospitalized definite or probable myocardial infarction (MI), definite fatal CHD, or silent MI. Definite fatal CHD was based on chest pain, CHD history, underlying cause of death from the death certificate, and other information from hospitalizations, medical histories, and ARIC visits. Silent MI was based on Minnesota-coded serial ECG changes over ARIC visits [29]. Definite or probable stroke events were based on signs, symptoms, neuroimaging, and other diagnostic reports according to National Survey of Stroke criteria [30, 31].
MI definition	Definite or probable MI was based on chest pain symptoms, electrocardiograms, and cardiac biomarkers from hospitalizations.
Surveillance Method	Incident CHD and stroke were ascertained among participants without history of CHD or stroke at baseline by contacting them annually, identifying hospitalizations and deaths during the previous year, surveying discharge lists from local hospitals, surveying death certificates from state vital statistics offices, and obtaining electrocardiograms (ECGs) at ARIC visits [28].
Medication ascertainment methods:	Medication Inventory at 4 Triennial Visits

Cardiovascular Health Study (CHS):

Methods for phenotype ascertainment for CHS has been described elsewhere [32, 33]; a summary is provided below.

Exclusions	Baseline history of myocardial infarction, stroke, or heart failure.<br>Missing genotyping results.
CVD definition	Definite, probable, or HCFA probable myocardial infarction, not procedure-related; Definite, probable, or HCFA probable stroke, not procedure-related; Heart failure; or Coronary heart disease death.
MI definition	Definite, probable, or HCFA probable myocardial infarction, not procedure-related.
Surveillance Method	CHS subjects were contacted every 6 months by either an in-person interview or by phone to assess CVD risk factors and occurrence of CVD events or hospitalizations. Discharge summaries and diagnoses were obtained for all hospitalizations, and all potential CVD events were reviewed and classified by an adjudication committee.
Medication ascertainment methods:	Subjects brought all medications to the yearly in-person interview, and medication use was assessed at that time. Once the yearly in-person interviews ceased, medication use was obtained yearly by phone interview.

Framingham Heart Study (FHS):

Methods for phenotype ascertainment for FHS has been described elsewhere [4, 7]; a summary is provided below.

Exclusions	Subjects who experienced MI, stroke, CHF at or prior to baseline exams were excluded from the study.
CVD definition	Incidence of MI, stroke (excluding transient ischemic attacks) or sudden death after the baseline exam.
MI definition	MI recognized, with diagnostic ECG; or MI recognized, without diagnostic ECG, but with enzymes and history.
Surveillance Method	An Endpoint Committee consisting of three physicians reviewed all suspected cardiovascular disease events to confirm occurrence of the event, using previously agreed criteria.
Medication ascertainment methods:	Questionnaire/interview at all exams except Exam 8 of the Offspring where study participants brought their medications to the exam and medications were recorded in a database using ATC codes.

Health ABC:

A summary of methods for phenotype ascertainment for Health ABC is provided below.

Exclusions	Prevalent MI, stroke, or heart failure. Not treated for hypertension.
CVD definition	MI, fatal CHD, or stroke. MI definition below. Fatal CHD definition is when the underlying cause of death is definite fatal MI or definite fatal CHD. Stroke definition is based on hospital diagnosis.
MI definition	At least one of the following: evolving diagnostic ECG pattern, diagnostic ECG pattern and abnormal enzymes, or cardiac pain or ischemic symptoms and abnormal enzymes with either an evolving ST-T pattern or an equivocal ECG pattern.
Surveillance Method	Hospitalization events were adjudicated using hospital records.
Medication ascertainment methods:	Medication inventory form, ingredients are mapped to medications using the IDIS dictionary for consistency with the WHAS data.

Heart and Vascular Health Study (HVH):

Methods for phenotype ascertainment for HVH has been described elsewhere [10]; a summary is provided below.

Exclusions	Prior history of a myocardial infarction, stroke, or heart failure at index.<br>Not using anti-hypertensive agents at index. Missing genotyping results.
CVD definition	First fatal or non-fatal myocardial infarction, or first fatal or non-fatal stroke, or sudden death event. Events are identified through ICD-9 and ICD-10 diagnostic codes from hospital discharge or from the Washington State death registry file. Trained abstractors review the Group Health (GH) medical records of the identified subjects to determine if a true event occurred.
MI definition	First fatal or non-fatal myocardial infarction. Events are identified through ICD-9/10 diagnostic codes from hospital discharge or from the Washington State death registry file and validated by trained abstractors.
Surveillance Method	Study participants are members of Group Health (GH), a large integrated health care system in western Washington State. Cases were identified from hospital discharge diagnosis codes and validated by medical record review. Controls were a random sample of GH members frequency matched to myocardial infarction cases on age (within decade), sex, treated hypertension, and calendar year of identification. Eligibility and risk factor information was collected using medical records reviews and telephone interviews.
Medication ascertainment methods:	Medication use was ascertained using computerized GH pharmacy records.

Jackson Heart Study (JHS):

Methods for phenotype ascertainment for JHS has been described elsewhere [14]; a summary is provided below.

Exclusions	ARIC–JHS Overlap
CVD definition	Self-reported history of myocardial infarction, coronary re-vascularization, or heart failure.
MI definition	Self-reported history of myocardial infarction.
Surveillance Method	N/A
Medication ascertainment methods:	Participants presented all of the medications used within 2 weeks whether prescriptions, over the counter or herbal preparations during clinical visit. [15] The Medi-Span® therapeutic classification system was used to identify medications. A registered pharmacist resolved and adjudicated any automated coding that led to indeterminate results.

Multi-Ethnic Study of Atherosclerosis (MESA):

Methods for phenotype ascertainment for MESA has been described elsewhere [34]; a summary is provided below.

Exclusions	No previous history of MI and CVD
CVD definition	Coronary heart disease (CHD), stroke (fatal or nonfatal), or other atherosclerotic CVD death
MI definition	Combination of chest pain, cardiac enzymes, and ECGs
Surveillance Method	Self-report, medical records and death certificates, interviews, questionnaires
Medication ascertainment methods:	Medication inventory

PROspective Study of Pravastatin in the Elderly at Risk (PROSPER):

Methods for phenotype ascertainment for PROSPER has been described elsewhere [18, 19]; a summary is provided below.

Exclusions	Subjects without treated hypertension
CVD definition	Combination of non-fatal and fatal MI and fatal and non-fatal Stroke
MI definition	Coronary heart disease death and definite & suspect non-fatal myocardial infarction
Surveillance Method	Endpoint committee
Medication ascertainment methods:	Oral communication

Rotterdam Study:
Methods for phenotype ascertainment for RS has been described elsewhere [35]; a summary is provided below.

Exclusions	Participants with missing genotype information, or missing information on drug use on the preceding research center visit
CVD definition	Incident myocardial infarction and stroke.
MI definition	Myocardial infarction was defined as a reported myocardial infarction with hospital admission or the presence of a myocardial infarction on the ECG. More details on methods of follow up and event adjudication have been described previously [22].
Surveillance Method	Rotterdam Study participants are continuously followed-up by direct digital linkage of the study base with medical files from the collaborating general practitioners. Subsequently two independent research physicians and either an experienced cardiologist or neurologist checked each potential event. Linkage to general practitioner databases, research center visits every 3–4 years.
Medication ascertainment methods:	Complete information on prescriptions for all participants was obtained in automated format from collaborating pharmacies.

Genetics of Hypertension Associated Treatment (GenHAT) study:

Methods for phenotype ascertainment for GenHAT has been described elsewhere [23, 25]; a summary is provided below.

Exclusions	n/a
CVD definition	Combined endpoint of incident CHD (fatal CHD and nonfatal MI) and stroke
MI definition	Incident fatal or nonfatal MI
Surveillance Method	Outcomes were reported by clinical investigators, and documentation (death certificate and hospital discharge summary) was submitted for any outcome involving death or hospitalization. National databases (Center for Medicare and Medicaid Services, the Department of Veteran's Affairs, the National Death Index, and the Social Security Administration) were also used to identify deaths occurring among participants lost to follow-up.
Medication ascertainment methods:	Randomized trial: Treatment was given once daily: chlorthalidone (12.5 mg for the first and second titration and 25 mg for the third), lisinopril (10, 20, or 40 mg), amlodipine (2.5, 5, or 10 mg), or doxazosin (2, 4, or 8 mg). If BP control was not achieved on the maximum study medication dose, a second-step, open-label agent (reserpine, clonidine, or atenolol) and/or a third-step open-label agent (hydralazine) was added. Information on study drug continuation, study drug cross-over, add-on antihypertensive therapy was recorded at yearly visits.

For this analysis drug exposures were assigned to the following categories:

- ACE: Randomized to ACE inhibitor and treated with ACE inhibitor during the year before the event, randomized to ACE inhibitor and missing drug crossover information the year before the event, or crossover to ACE inhibitor treatment the year before the event.
- Calcium Channel Blocker (CCB): Randomized to CCB and treated with CCB during the year before the event, randomized to CCB and missing drug crossover information the year before the event, or crossover to CCB treatment the year before the event.
- Thiazide Diuretic (TD): Randomized to TD and treated with TD during the year before the event, randomized to TD and missing drug crossover information the year before the event, or crossover to TD treatment the year before the event.
- Reference: Treated with a study drug or add-on drug not of the exposure category the year before the event.

Note: If any participant was on some combination of ACE/TD/CCB they were "exposed" in each analysis; if none of the drugs included the "exposure" for that analysis, they were included in the reference group.

Section C: Study-specific genotyping and imputation methods

	Genotyping		Sample QC		SNP QC		Imputation		
	Platform	Calling	Call rate	Other exclusions	Call freq.	Other exclusions	N SNPs	Software	Reference panel
**AGES**	Illumina Hu370CNV	Illumina BeadStudio	< 95%	Discordant with prior Illumina genotyping (OPA)	< 90%	HWE $p < 10^{-6}$ bad position	326,034	MaCH	HapMap P2.r22.b36, CEU
**ARIC-EA**	Affymetrix 6.0	Birdseed	< 95%	Sex discrepancy, Race discrepancy, Duplicate Sample 1° Relative, Sample Handling Error, No Principal Components, No Imputed Data, Known ALLHAT Participants	< 90%	MAF < 1% HWE $p < 10^{-5}$	597,357	MacH	HapMap P2.r22.b36, CEU
**ARIC-AA**	Affymetrix 6.0	Birdseed	< 95%		< 90%	MAF < 1%	645,974	MaCH	HapMap P2.r22.b36, 1:1 CEU:YRI
**CHS-EA**	Illumina Hu370CNV	Illumina BeadStudio	< 95%	Sex discrepancy Discordant with prior genotyping	< 97%	HWE $P < 10^{-5}$ > 2 DE/MeI hz frequency = 0, not in HapMap	306,655	BIMBAM	HapMap P2.r22.b36, CEU
**CHS-AA**	Illumina Human1M-Duo	Illumina GenomeStudio	< 95%		< 97%	HWE $P < 10^{-5}$, > 1 DE/MeI hz frequency = 0.	940,567	BEAGLE	HapMap P2.r22.b36, (CEU, YRI) & HapMap Phase 3 (YRI, ASW, CEU)
**FHS**	Affymetrix 500K (Nsp 250K and Sty 250K) and MIPS 50K combined	BRLMM	< 97%	Excess heterozygosity (5 SD from the mean); > 1000 Mendelian errors	< 97%	MAF<0.01; HWE $p<10^{-6}$; mishap $p<10^{-9}$; >100 MeI; strand issues or not in HapMap	378,163	MaCH	HapMap P2.r22.b36, CEU
**Health ABC**	Illumina Human1M-Duo	Illumina BeadStudio	≤ 97%	Sex discrepancy 1° Relative	< 97%	MAF < 0.01 HWE $p < 10^{-6}$ Not present in HapMap	914,263	MaCH	HapMap P2.r22.b36, CEU
**HVH1**	Illumina Hu370CNV	Illumina BeadStudio	< 95%	Sex discrepancy	< 97%	HWE $P < 10^{-5}$ > 2 DE/MeI hz frequency = 0, SNP not in HapMap	301,321	BIMBAM	HapMap P2.r22.b36, CEU
**HVH2**	Illumina Omni Express	Illumina GenomeStudio	< 95%		< 97%	HWE $P < 10^{-5}$ > 2 DE/MeI hz frequency = 0, SNP not in HapMap	694,303	MaCH	HapMap P2.r22.b36, CEU
**JHS**	Affymetrix 6.0	Birdseed	< 95%	Sample duplicates, contaminated samples, excess heterozygosity, cryptic relatedness, sample outliers	<90%	MAF < 0.01 HWE $p < 10^{-6}$, MeI , mapping to several genomic locations	868,969	MaCH	HapMap P2.r22.b36, CEU+YRI
**MESA-EA**	Affymetrix 6.0	Birdseed	< 95%	Sex discrepancy Cryptic duplicates	< 95%	MAF < 0.1 HWE $p < 10^{-4}$	849,709	IMPUTE	HapMap P2.r24.b36, CEU
**MESA-AA**	Affymetrix 6.0	Birdseed	< 95%		< 95%	MAF < 0.1 HWE $p < 10^{-4}$	849,709	IMPUTE	HapMap P2.r24.b36, CEU+YRI+CHB+JPT
**PROSPER**	Illumina 660K	Illumina BeadStudio	< 97.5%	Sex discrepancy ,Cryptic duplicates ,Familial relationships, Excess heterozygosity, non-EA	< 95%	HWE $p < 10^{-6}$	557,192	MaCH	HapMap P2.r22.b36, CEU
**RS**	Illumina HumanHap550 v3.0	Illumina BeadStudio	< 98%	Sex discrepancy Excess heterozygosity, Outliers by IBS clustering	<98%	HWE p <10-6	512,349	MaCH	HapMap P2.r22.b36, CEU

Abbreviations in table: MAF, minor allele frequency; HWE, Hardy-Weinberg Equilibrium; DE, duplicate errors; MeI, Mendelian inconsistencies; hz=heterozygote; P2.r22.b36, Phase II, release 22, build 36

Section D: Study-specific analysis methods

Study	Adjustments	Analysis method	Software
AGES	Age, sex	Cox Regression	R (version 2.15.2)
ARIC	Age, sex,<br>Study site<br>Principal components (3 for EAs; 10 for AAs)	Cox Regression	SAS(R) 8.2<br>PROC PHREG
CHS	Age, sex,<br>Study site,<br>PCs (0 for EAs; 5 for AAs)	Cox regression	R
FHS	Age, sex,<br>cohort,<br>calendar year (event date for cases and calendar year for controls),<br>PCs of population structure that were significantly associated with event status.	Logistic regression	R version 2.9.2<br>GEE in *geepack*
Health ABC	Age, sex,<br>Study site,<br>principal components	Cox regression	R version 2.15
HVH	age, sex,<br>index year (grouped by OR to minimize number of adjustment variables)	Logistic regression	R
JHS	Age, sex,<br>10 PCs	Logistic regression	ProbABEL
MESA	Age, sex,<br>PCs	Cox Regression	R
PROSPER	Age, sex,<br>country of origin	Logistic regression	ProbABEL
RS	Age, sex	Logistic regression	ProbABEL, R
GENHAT	None	Logistic regression	PLINK

Software URLs:

PROBABEL: http://www.genabel.org/
R: http://cran.r-project.org/
SAS: http://www.sas.com

References

1. Harris TB, Launer LJ, Eiriksdottir G, Kjartansson O, Jonsson PV, Sigurdsson G, et al. Age, Gene/Environment Susceptibility-Reykjavik Study: multidisciplinary applied phenomics. Am J Epidemiol. 2007;165(9):1076-87. Epub 2007/03/14. doi: kwk115 [pii]

10.1093/aje/kwk115. PubMed PMID: 17351290.

2. The Atherosclerosis Risk in Communities (ARIC) Study: design and objectives. The ARIC investigators. Am J Epidemiol. 1989;129(4):687-702. Epub 1989/04/01. PubMed PMID: 2646917.

3. Fried LP, Borhani NO, Enright P, Furberg CD, Gardin JM, Kronmal RA, et al. The Cardiovascular Health Study: design and rationale. Ann Epidemiol. 1991;1(3):263-76. PubMed PMID: 1669507.

4. Dawber TR, Kannel WB, Lyell LP. An approach to longitudinal studies in a community: the Framingham Study. Ann N Y Acad Sci. 1963;107:539-56. Epub 1963/05/22. PubMed PMID: 14025561.

5. Dawber TR, Meadors GF, Moore FE, Jr. Epidemiological approaches to heart disease: the Framingham Study. American journal of public health and the nation's health. 1951;41(3):279-81. Epub 1951/03/01. PubMed PMID: 14819398; PubMed Central PMCID: PMC1525365.

6. Feinleib M, Kannel WB, Garrison RJ, McNamara PM, Castelli WP. The Framingham Offspring Study. Design and preliminary data. Prev Med. 1975;4(4):518-25. Epub 1975/12/01. PubMed PMID: 1208363.

7. Kannel WB, Feinleib M, McNamara PM, Garrison RJ, Castelli WP. An investigation of coronary heart disease in families. The Framingham offspring study. Am J Epidemiol. 1979;110(3):281-90. Epub 1979/09/01. PubMed PMID: 474565.

8. Splansky GL, Corey D, Yang Q, Atwood LD, Cupples LA, Benjamin EJ, et al. The Third Generation Cohort of the National Heart, Lung, and Blood Institute's Framingham Heart Study: design, recruitment, and initial examination. Am J Epidemiol. 2007;165(11):1328-35. Epub 2007/03/21. doi: kwm021 [pii]

10.1093/aje/kwm021. PubMed PMID: 17372189.

9. Psaty BM, Heckbert SR, Atkins D, Lemaitre R, Koepsell TD, Wahl PW, et al. The risk of myocardial infarction associated with the combined use of estrogens and progestins in postmenopausal women. Arch Intern Med. 1994;154(12):1333-9. Epub 1994/06/27. PubMed PMID: 8002685.

10. Psaty BM, Heckbert SR, Koepsell TD, Siscovick DS, Raghunathan TE, Weiss NS, et al. The risk of myocardial infarction associated with antihypertensive drug therapies. Jama. 1995;274(8):620-5. PubMed PMID: 7637142.

11. Klungel OH, Heckbert SR, Longstreth WT, Jr., Furberg CD, Kaplan RC, Smith NL, et al. Antihypertensive drug therapies and the risk of ischemic stroke. Arch Intern Med. 2001;161(1):37-43. Epub 2001/01/09. PubMed PMID: 11146696.

12. Lemaitre RN, King IB, Rice K, McKnight B, Sotoodehnia N, Rea TD, et al. Erythrocyte very long-chain saturated fatty acids associated with lower risk of incident sudden cardiac arrest. Prostaglandins, leukotrienes, and essential fatty acids. 2014;91(4):149-53. Epub 2014/08/12. doi: 10.1016/j.plefa.2014.07.010. PubMed PMID: 25107579; PubMed Central PMCID: PMC4156887.

13. Fuqua SR, Wyatt SB, Andrew ME, Sarpong DF, Henderson FR, Cunningham MF, et al. Recruiting African-American research participation in the Jackson Heart Study: methods, response rates, and sample description. Ethnicity & disease. 2005;15(4 Suppl 6):S6-18-29. Epub 2005/12/02. PubMed PMID: 16317982.

14. Keku E, Rosamond W, Taylor HA, Jr., Garrison R, Wyatt SB, Richard M, et al. Cardiovascular disease event classification in the Jackson Heart Study: methods and procedures. Ethnicity & disease. 2005;15(4 Suppl 6):S6-62-70. Epub 2005/12/02. PubMed PMID: 16317987.

15. Harman J, Walker ER, Charbonneau V, Akylbekova EL, Nelson C, Wyatt SB. Treatment of hypertension among African Americans: the Jackson Heart Study. Journal of clinical hypertension. 2013;15(6):367-74. Epub 2013/06/05. doi: 10.1111/jch.12088. PubMed PMID: 23730984; PubMed Central PMCID: PMC3683967.

16. Musunuru K, Lettre G, Young T, Farlow DN, Pirruccello JP, Ejebe KG, et al. Candidate gene association resource (CARe): design, methods, and proof of concept. Circ Cardiovasc Genet. 2010;3(3):267-75. Epub 2010/04/20. doi: 10.1161/CIRCGENETICS.109.882696. PubMed PMID: 20400780; PubMed Central PMCID: PMC3048024.

17. Bild DE, Bluemke DA, Burke GL, Detrano R, Diez Roux AV, Folsom AR, et al. Multi-ethnic study of atherosclerosis: objectives and design. Am J Epidemiol. 2002;156(9):871-81. Epub 2002/10/25. PubMed PMID: 12397006.

18. Shepherd J, Blauw GJ, Murphy MB, Cobbe SM, Bollen EL, Buckley BM, et al. The design of a prospective study of Pravastatin in the Elderly at Risk (PROSPER). PROSPER Study Group. PROspective Study of Pravastatin in the Elderly at Risk. Am J Cardiol. 1999;84(10):1192-7. Epub 1999/11/24. PubMed PMID: 10569329.

19. Shepherd J, Blauw GJ, Murphy MB, Bollen EL, Buckley BM, Cobbe SM, et al. Pravastatin in elderly individuals at risk of vascular disease (PROSPER): a randomised controlled trial. Lancet. 2002;360(9346):1623-30. Epub 2002/11/30. PubMed PMID: 12457784.

20. Trompet S, de Craen AJ, Postmus I, Ford I, Sattar N, Caslake M, et al. Replication of LDL GWAs hits in PROSPER/PHASE as validation for future (pharmaco)genetic analyses. BMC Med Genet. 2011;12:131. Epub 2011/10/08. doi: 10.1186/1471-2350-12-131. PubMed PMID: 21977987; PubMed Central PMCID: PMC3207930.

21. Hofman A, Breteler MM, van Duijn CM, Krestin GP, Pols HA, Stricker BH, et al. The Rotterdam Study: objectives and design update. Eur J Epidemiol. 2007;22(11):819-29. Epub 2007/10/24. doi: 10.1007/s10654-007-9199-x. PubMed PMID: 17955331.

22. Leening MJ, Kavousi M, Heeringa J, van Rooij FJ, Verkroost-van Heemst J, Deckers JW, et al. Methods of data collection and definitions of cardiac outcomes in the Rotterdam Study. Eur J Epidemiol. 2012;27(3):173-85. Epub 2012/03/06. doi: 10.1007/s10654-012-9668-8. PubMed PMID: 22388767; PubMed Central PMCID: PMC3319884.

23. Davis BR, Cutler JA, Gordon DJ, Furberg CD, Wright JT, Jr., Cushman WC, et al. Rationale and design for the Antihypertensive and Lipid Lowering Treatment to Prevent Heart Attack Trial (ALLHAT). ALLHAT Research Group. Am J Hypertens. 1996;9(4 Pt 1):342-60. Epub 1996/04/01. PubMed PMID: 8722437.

24. Arnett DK, Boerwinkle E, Davis BR, Eckfeldt J, Ford CE, Black H. Pharmacogenetic approaches to hypertension therapy: design and rationale for the Genetics of Hypertension Associated Treatment (GenHAT) study. Pharmacogenomics J. 2002;2(5):309-17. Epub 2002/11/20. doi: 10.1038/sj.tpj.6500113. PubMed PMID: 12439737.

25. Major cardiovascular events in hypertensive patients randomized to doxazosin vs chlorthalidone: the antihypertensive and lipid-lowering treatment to prevent heart attack trial (ALLHAT). ALLHAT Collaborative Research Group. JAMA. 2000;283(15):1967-75. Epub 2000/05/02. PubMed PMID: 10789664.

26. Lynch AI, Irvin MR, Boerwinkle E, Davis BR, Vaughan LK, Ford CE, et al. RYR3 gene polymorphisms and cardiovascular disease outcomes in the context of antihypertensive treatment. Pharmacogenomics J. 2013;13(4):330-4. Epub 2012/06/06. doi: 10.1038/tpj.2012.22. PubMed PMID: 22664477; PubMed Central PMCID: PMC3435442.

27. Major outcomes in high-risk hypertensive patients randomized to angiotensin-converting enzyme inhibitor or calcium channel blocker vs diuretic: The Antihypertensive and Lipid-Lowering Treatment to Prevent Heart Attack Trial (ALLHAT). Jama. 2002;288(23):2981-97. Epub 2002/12/20. PubMed PMID:

12479763.

28. White AD, Folsom AR, Chambless LE, Sharret AR, Yang K, Conwill D, et al. Community surveillance of coronary heart disease in the Atherosclerosis Risk in Communities (ARIC) Study: methods and initial two years' experience. Journal of clinical epidemiology. 1996;49(2):223-33. Epub 1996/02/01. PubMed PMID: 8606324.

29. Prineas RJ, Crow RS, Blackburn HW. The Minnesota code manual of electrocardiographic findings : standards and procedures for measurement and classification. 2nd ed. London: Springer; 2010. xiii, 328 p. p.

30. Rosamond WD, Folsom AR, Chambless LE, Wang CH, McGovern PG, Howard G, et al. Stroke incidence and survival among middle-aged adults: 9-year follow-up of the Atherosclerosis Risk in Communities (ARIC) cohort. Stroke. 1999;30(4):736-43. Epub 1999/04/03. PubMed PMID: 10187871.

31. The National Survey of Stroke. National Institute of Neurological and Communicative Disorders and Stroke. Stroke. 1981;12(2 Pt 2 Suppl 1):I1-91. Epub 1981/03/01. PubMed PMID: 7222163.

32. Ives DG, Fitzpatrick AL, Bild DE, Psaty BM, Kuller LH, Crowley PM, et al. Surveillance and ascertainment of cardiovascular events. The Cardiovascular Health Study. Ann Epidemiol. 1995;5(4):278-85. PubMed PMID: 8520709.

33. Price TR, Psaty B, O'Leary D, Burke G, Gardin J. Assessment of cerebrovascular disease in the Cardiovascular Health Study. Ann Epidemiol. 1993;3(5):504-7. PubMed PMID: 8167827.

34. Detrano R, Guerci AD, Carr JJ, Bild DE, Burke G, Folsom AR, et al. Coronary calcium as a predictor of coronary events in four racial or ethnic groups. N Engl J Med. 2008;358(13):1336-45. Epub 2008/03/28. doi: 10.1056/NEJMoa072100. PubMed PMID: 18367736.

35. Hofman A, Darwish Murad S, van Duijn CM, Franco OH, Goedegebure A, Ikram MA, et al. The Rotterdam Study: 2014 objectives and design update. Eur J Epidemiol. 2013;28(11):889-926. Epub 2013/11/22. doi: 10.1007/s10654-013-9866-z. PubMed PMID: 24258680.
